# Crystal Structure Survey and Theoretical Analysis of Bifurcated Halogen Bonds

**DOI:** 10.1021/acs.cgd.2c00726

**Published:** 2022-10-10

**Authors:** Mariusz Michalczyk, Wiktor Zierkiewicz, Steve Scheiner

**Affiliations:** † Faculty of Chemistry, Wrocław University of Science and Technology, Wybrzeże Wyspiańskiego 27, 50-370 Wrocław, Poland; ‡ Department of Chemistry and Biochemistry, Utah State University Logan, Logan, Utah 84322-0300, United States

## Abstract

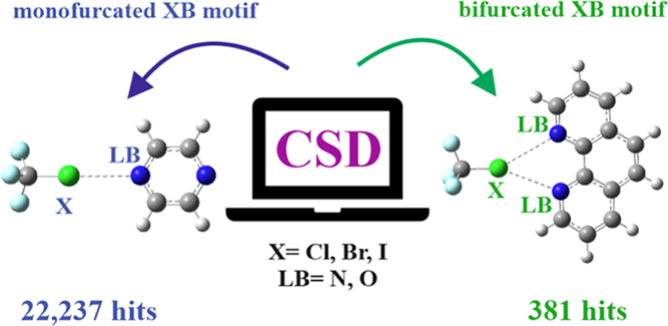

The possibility that two Lewis bases can share a single halogen atom within the context of a bifurcated halogen bond (XB) is explored first by a detailed examination of the CSD. Of the more than 22,000 geometries that fit the definition of an XB (with X = Cl, Br, I), less than 2% are bifurcated. There is a heavy weighting of I in such bifurcated arrangements as opposed to Br, which prefers monofurcated bonds. The conversion from mono to bifurcated is associated with a smaller number of short contact distances, as well as a trend toward lesser linearity. The two XBs within a bifurcated system are somewhat symmetrical: the two lengths generally differ by less than 0.05 Å, and the two XB angles are within several degrees of one another. Quantum calculations of model systems reflect the patterns observed in crystals and reinforce the idea that the negative cooperativity within a bifurcated XB weakens and lengthens each individual bond.

## Abstract 2

The possibility that two Lewis bases can share a single halogen atom within the context of a bifurcated halogen bond (XB) is explored first by a detailed examination of the CSD. Of the more than 22,000 geometries that fit the definition of an XB (with X = Cl, Br, I), less than 2% are bifurcated. There is a heavy weighting of I in such bifurcated arrangements as opposed to Br, which prefers monofurcated bonds. The conversion from mono to bifurcated is associated with a smaller number of short contact distances, as well as a trend toward lesser linearity. The two XBs within a bifurcated system are somewhat symmetrical: the two lengths generally differ by less than 0.05 Å, and the two XB angles are within several degrees of one another. Quantum calculations of model systems reflect the patterns observed in crystals and reinforce the idea that the negative cooperativity within a bifurcated XB weakens and lengthens each individual bond.

## Introduction

It has long been understood that the preferred geometry of a H-bond is linear, in that the bridging proton prefers to lie directly along the axis connecting the proton donor and acceptor atoms.^1−8^ This arrangement is of course, not absolutely mandatory, and the H-bond can be bent by forces external to it, as in an intramolecular system or within the context of the many packing forces in a crystal environment. An interesting wrinkle on the H-bond concept arises when two electron donors both approach the proton donor from somewhat different directions, forming what is commonly referred to as a bifurcated H-bond.^9−11^ The benefit of forming a second H-bond must be weighed against the disadvantage that both of these interactions are, of necessity, nonlinear. A second factor is the negative cooperativity that arises when the proton donor group serves simultaneously as a double electron acceptor.

Bifurcated H-bonds are rather common, as revealed by numerous surveys of crystal structures.^12^ Such configurations have generated a great deal of study over the years.^10,13−17^ The 2-fluoroethanol trimer^11^ represents one particular example, where a single H atom can engage with O and N atoms simultaneously.^18^ An instance drawn from biology^19^ occurs within the α-helix N-Caps of ankyrin repeat proteins. Bifurcated H-bonds are also necessary to stabilize fibrils of poly(l-glutamic) acid.^20^ Another example^21^ places a halide between two H atoms of an alkenic = CH_2_ group.

Halogen bonds (XBs) have much in common with H-bonds.^22−42^ The replacement of the bridging proton by a halogen atom leaves intact much of the underlying phenomena that contribute to their stability. While the bridging X atom does not carry an overall positive charge as does H, the strong anisotropy of the surrounding electron cloud leads to a small region of positive charge directly along the extension of the R–X covalent bond, commonly dubbed a σ-hole. Due to their similarity to H-bonds, it is not surprising that bifurcated XBs occur and have undergone some scrutiny.^43−45^ As one example, there is recent evidence of a bifurcated XB^46^ with a C-Br group interacting with both Cl and Pt atoms. In another example, an aryl I atom participates^47^ in a bifurcated XB with a pair of O atoms of a PtO_4_ unit. An I atom of a PtI_2_Br_2_ moiety^48^ is involved in several halogen bonds simultaneously. A halogen atom placed between two N atoms in a bidentate diazaheterocyclic compound^49^ provides another example. Even the first-row F halogen appears capable of participating in a bifurcated halogen bond,^50^ albeit under certain conditions. A recent computational study^51^ showed that a pair of N bases preferred to be separated by an intermolecular θ(N···X···N) angle of 60° when placed near the halogen atom of FBr or FI. This bifurcated XB is much less stable than the monofurcated linear XB with a single base, and the R(X···N) distances are somewhat longer. While much is now understood about the fundamental properties of a stand-alone XB, the study of the corresponding bifurcated XBs remains in its infancy.

This work is intended to place bifurcated XBs in their proper context. The CSD is surveyed first to determine their prevalence and how likely one might be observed in comparison with a standard monofurcated XB. As a subtopic, does the likelihood of a bifurcated XB vary with the identity of the particular halogen atom? There are certain geometric tendencies of an XB, for example, a propensity toward linearity and a shortening of the intermolecular distance relative to the sum of vdW radii. How might these preferences change when the X atom must accommodate two electron donor groups? Are the two bases symmetrically disposed around the X, or is there a clear geometric distinction between them? To better understand the underlying causes for these observations, a series of model systems, both mono- and bifurcated XBs, are subjected to quantum chemical inquiry. These calculations pinpoint the way in which the intrinsic geometric preferences of each sort of bond change when a second base is added and how these properties stand up to the imposition of crystal packing forces. The calculations also show how these forces are guided by energetic considerations, i.e., what is the potential benefit of adding a second base to an XB.

## Computational Details

Solid-state geometries were accessed through the Cambridge Structural Database (CSD, ver. 5.42 with updates) and supporting CCDC software Mercury and ConQuest.^52,53^ These programs were employed for the statistical analysis of the CSD findings as well. Quantum calculations were performed at the PBE0-D3/def2TZVP^54−56^-level of theory. Calculations invoked structures from the CSD directly with no optimization. The theoretical models of idealized mono- and bifurcated complexes were fully optimized with no constraints, along with harmonic frequency analysis to confirm these structures represent true minima. Interaction energies referred to the difference between the complex and the sum of monomers, all in the geometry within the optimized complex. The basis set superposition error (BSSE) was removed via the counterpoise procedure introduced by Boys and Bernardi.^57^ The quantum calculations were carried out within the framework of the Gaussian 16, Rev. C.01 set of codes.^58^ Bader’s QTAIM protocol^59^ provided analysis of the electron density topology by means of the AIMAll suite of programs.^60^

## Results

### CSD Survey

**Scheme 1:**
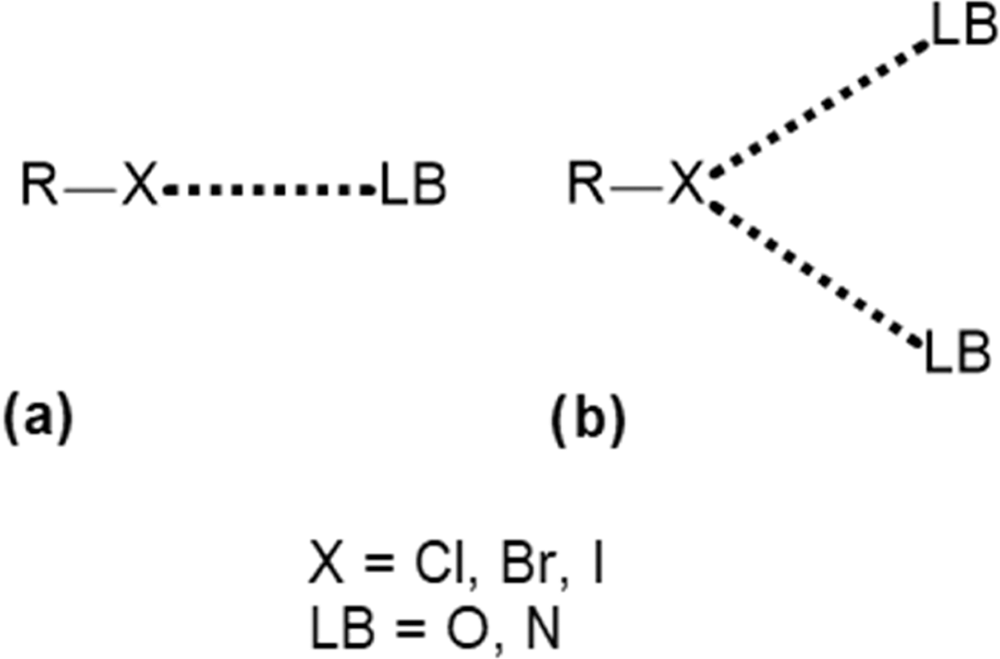
Two Possible Types of Halogen Bond Topology: (a) Mono, (b) Bifurcated Bonds

Bonds between a halogen atom (X = Cl, Br, I) and one or two or three atoms (either O or N) on a neighboring unit led to three separate surveys, one for each X. The criteria for identifying XBs within the CSD were as follows. The distance between X and O/N must be shorter than the Alvarez vdW radii sum.^61^ The R-X···O/N angle must lie in the 150–180° range. Additional technical restraints were applied in ConQuest software: only structures with 3D coordinates defined, nondisordered, with no errors, and with *R* < 0.1 were allowed in searches. The mono- and bifurcated XBs are illustrated in Scheme 1, along with the atoms considered to participate in each bond.

**Figure 1:**
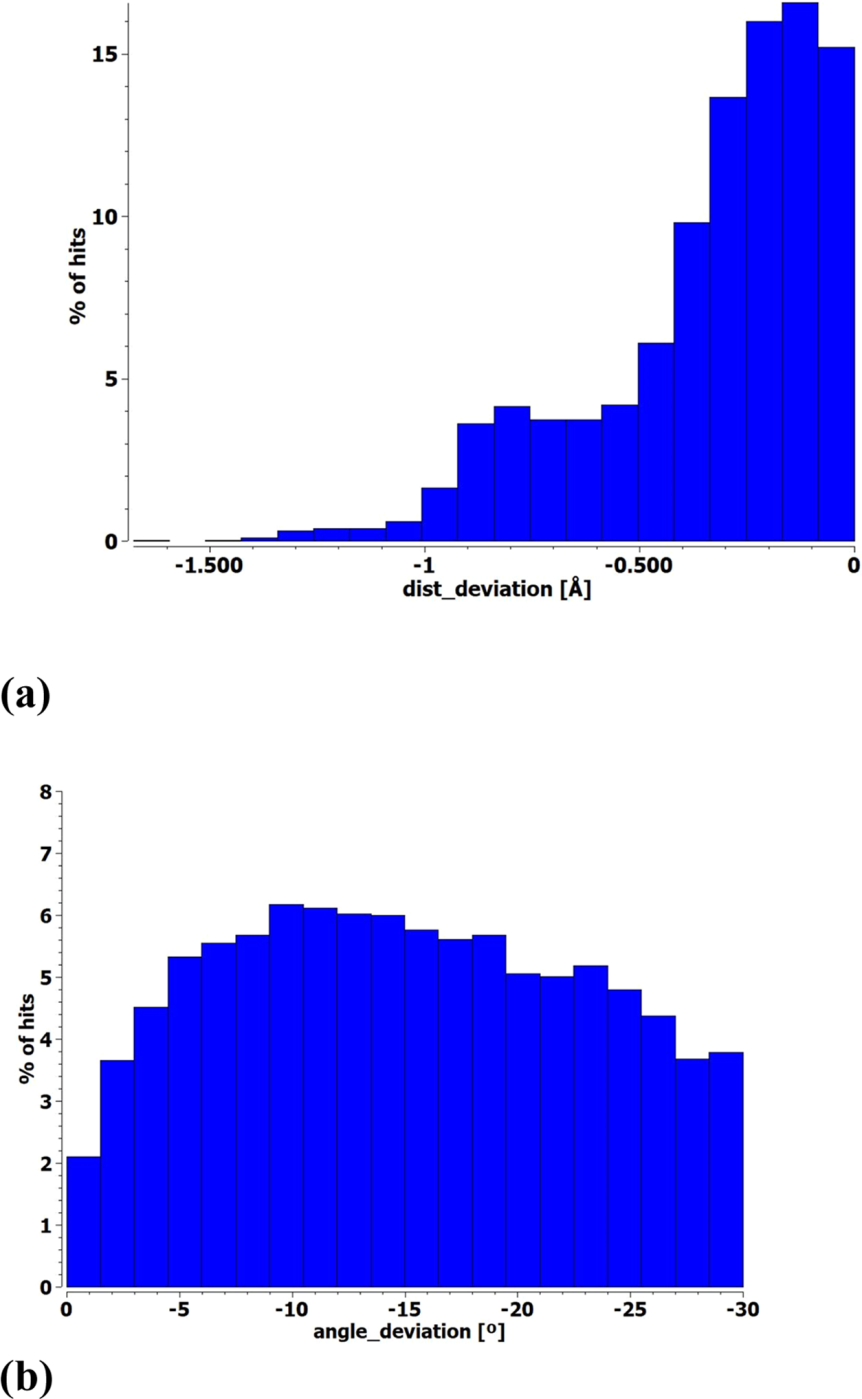
Histograms of percentage of hits of monofurcated XBs identified in CSD. The distance deviation in panel (a) refers to the difference versus the vdW radii sum, a different sum for each pair of atoms, and the angle deviation in panel (b) indicates the difference from linearity.

First, regarding simple monofurcated XBs with only one electron donor, there were a total of 22,237 hits. Such a large number is not unexpected due to the prevalence of the XB as a general phenomenon. Figure 1 represents a statistical report of these data in the form of histograms involving the XB length and angle. Specifically, Figure 1a plots the number of hits for each contraction of the X···O/N distance as compared to the vdW sum, so displacement to the left accords with a shorter XB. The deviation from linearity of the XB angle is displayed in Figure 1b, where the angle deviation refers to θ(RX···O/N) – 180°.

**Figure 2:**
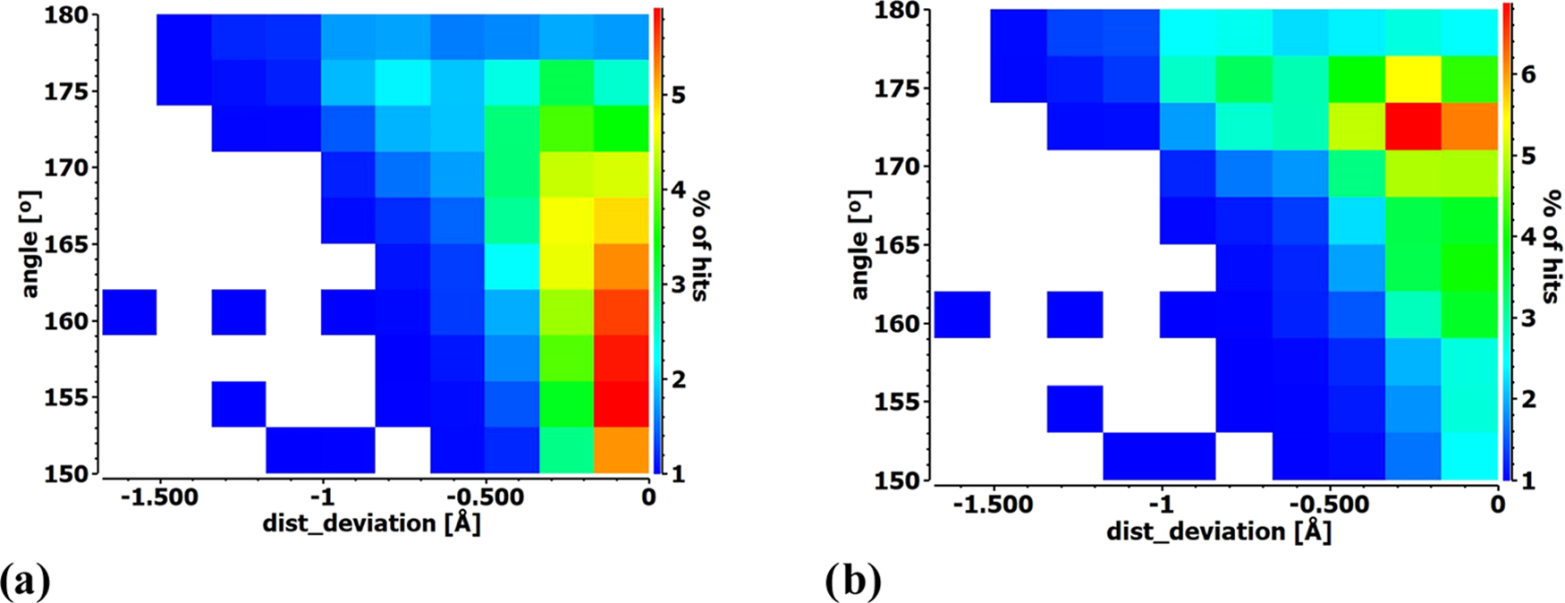
Heat plots presenting the number of hits for a single XB, with a particular θ(R-X···O/N) angle and the X···O/N distance, expressed as the deviation from the vdW radii sum for X = Cl, Br, I. The raw data in panel (a) is adjusted by the cone correction in panel (b). The number of hits is referenced to the overall number of found structures as the percentage contribution (as shown in the color scale bar).

The peak occurs at just under 10°. The relatively small number of occurrences closer to 180° is caused by geometric factors. To illustrate this point, Figure 2a contains a heat plot of this same data, where red indicates the largest proportion of particular geometries. The horizontal axis represents the contraction of the X···O/N distance, and the θ(RX···O/N) angle applies to the vertical axis. Regarding the angle, the largest grouping aggregate is in the 155–165° range, not quite the linearity that is predicted by ideas of a σ-hole or orbital overlap. On the other hand, there is a statistical bias toward smaller angles since the cone encompassing a given angle grows larger as θ diminishes from 180°. This bias can be erased by a so-called cone correction invoking the sinθ function.^62−65^ The corrected heat plot presented in Figure 2b shows that the preferred angle moves much closer to linearity, with the maximum proportion of hits between 170° and 175°.

A pie chart illustrating the percentages of each of the six variants of these halogen bonds is supplied in Figure S1. In brief, The largest fraction places Cl in contact with O, representing fully one-third of all such complexes. Indeed, the combination of Cl···O with Cl···N accounts for fully half of all of these bonds. One should not associate this preponderance with greater strength, since numerous works have shown that the XB strength rises with the size of X atom, so Cl XBs would tend to be weaker than others. The large number of such bonds is simply a result of the prevalence of Cl in systems for which crystals are available.

### Bifurcated XBs

**Figure 3:**
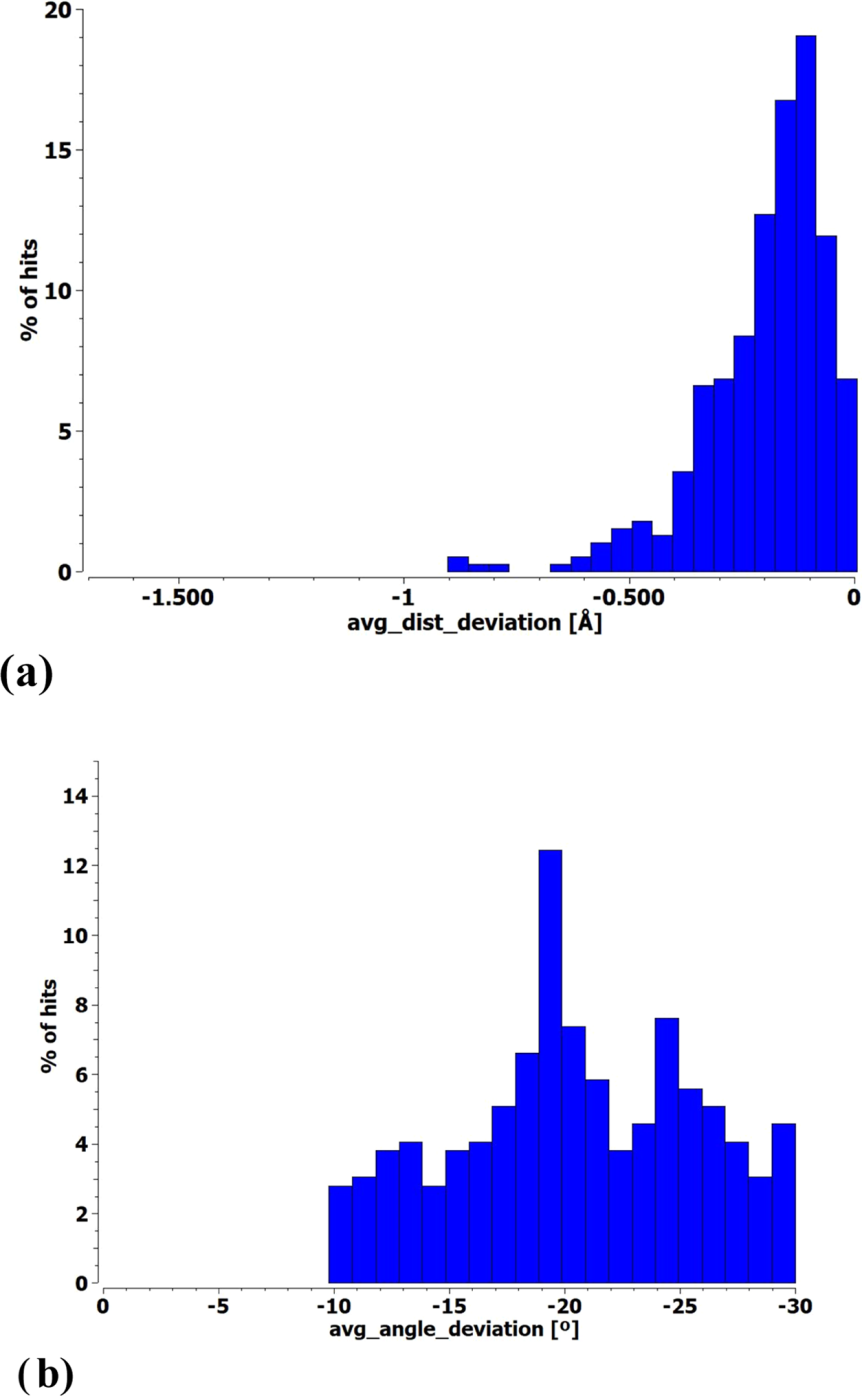
Histograms of percentage of hits of bifurcated XBs identified in CSD. The (a) distance deviation refers to the difference from the sum of vdW radii of each atom pair, and (b) angle deviations represent the mean of the two bonds.

Turning to bifurcated XBs, these were limited to systems with two homogenic X···N or X···O (X = Cl, Br, I) contacts within their vdW radii sum. In other words, mixed X···N with X···O were excluded, and F was not considered as an XB donor, as it has been shown to engage in such a bond only rarely and under extreme circumstances. Analysis provided 381 situations with clear bifurcated halogen bonding, with a pair of electron donors sharing a single X atom. The data are presented first as averages of the two XBs in the form of histograms in Figure 3.

With respect to the XB lengths, the addition of a second XB seems to bunch the data up toward the right, as seen in the comparison of Figure 3a with Figure 1a; there are fewer short bonds in the bifurcated case. This trend can be understood as negative cooperativity arising since two bases sharing the same X electron acceptor would tend to weaken and lengthen the XBs. The angular deviations from linearity in Figures 1b and 3b show a general displacement to the right toward nonlinearity. This trend is again sensible in view of the steric repulsions between any two bases. Even if one came close to a linear arrangement, the large ensuing displacement of the other base would give rise to a high average angular deviation.

The small fraction of bifurcated XBs vs their monofurcated counterparts is obvious from Table 1, less than 3% for any particular normalized bond length. With regard to the dependence of this fraction on the bond length, it seems to peak for bond contractions of about 0.20 Å but drops off for shorter bonds. Indeed, there is a marked deficiency of very short bifurcated XBs, even on a percentage basis.

**Table 1:** Number of Hits of Mono- and Bifurcated XBs Identified in CSD and Their Percentage Contribution

R–R_vdW_	monofurcated XBs	bifurcated XBs	bif/mono [%]
0.0 to **–**0.1	5658	101	1.8
**–**0.1 to **–**0.2	5383	137	2.5
**–**0.2 to **–**0.3	4191	72	1.7
**–**0.3 to **–**0.4	2744	41	1.5
**–**0.4 to **–**0.5	1300	14	1.1
**–**0.5 to **–**0.6	778	11	1.4
**–**0.6 to **–**0.7	601	1	0.2
**–**0.7 to **–**0.8	590	1	0.2
**–**0.8 to **–**0.9	526	2	0.4
**–**0.9 to **–**1.0	274	1	0.4
**–**1.0 to **–**1.1	72	0	0.0
**–**1.1 to **–**1.2	43	0	0.0
**–**1.2 to **–**1.3	59	0	0.0
**–**1.3 to **–**1.4	14	0	0.0
**–**1.4 to **–**1.5	4	0	0.0

The distance and angular factors are combined in the heat plot of Figure S2, which shows that the most common geometrical arrangement for this motif is represented by the red square (around 8% of all hits in the CSD survey). The situation to which this square corresponds has the average X···O/N distance shorter than the vdW sum by about 0.1–0.2 Å, and the average R-X···N/O angle deviates by 18–19° from linearity. (Note that no cone correction has been applied to Figures 3 and S2, as there were two angles involved in each configuration.)

With regard to the specific atoms involved in each XB, Figure S1b indicates that there is a strong reduction in Br···O XBs, diminishing from 20% to 8% on going from mono to bifurcated. Also there is a shift away from Cl···O to Cl···N. Perhaps the most dramatic change is the increase in I XBs. The proportion of these bonds rises from 21% of all monofurcated to 51% bifurcated XBs. In essence then, the preponderance of Cl monofurcated XBs shifts to I in the bifurcated mode. Also of particular importance are the numbers of each sort of XB, monofurcated vs bifurcated. The survey identified 381 bifurcated XBs, which compares with a sum of 22,237 XBs in all. In a statistical sense, then, less than 2% of XBs observed fall into the category of a bifurcated bond.

Another way in which to analyze the data concerns the propensity of each sort of XB to engage in bifurcated vs monofurcated geometry. Table 2 summarizes the number of hits for each type of mono- and bifurcated halogen bonds, followed by the percentage of this particular XB class to the total. So as to address the issue of the likelihood of bifurcation, the number of hits corresponding to a given type of bifurcated XB was divided by the total number of monofurcated halogen bonds. The results are shown in the last column of this table. First, each bond type accounts for less than 0.5% of all monofurcated XBs, emphasizing the generally low probability of bifurcation. Taking each atom pair individually, the largest numbers are those in which two nitrogen atoms are involved as electron donors, with iodine accounting for 0.5% and chlorine 0.4%. Second, the smallest fraction of structures with a bifurcated halogen bond involves Br, 0.1% each for the Br···N and Br···O bond types.

**Table 2:** Number of Hits of Mono- and Bifurcated XBs Identified in CSD and Their Percentage Contribution

	monofurcated XBs		bifurcated XBs		
bond type	no. found in CSD	% of all mono XBs	no. found in CSD	% of all bifurcated XBs	% of all mono XBs^a^
Cl···O	7338	33	34	9	0.2
Br···O	4447	20	31	8	0.1
I···O	2446	11	76	20	0.3
Cl···N	4003	18	95	25	0.4
Br···N	1779	8	27	7	0.1
I···N	2224	10	118	31	0.5

a Fraction of each sort of bond that is bifurcated compared to the total number of all monofurcated XBs.

**Figure 4:**
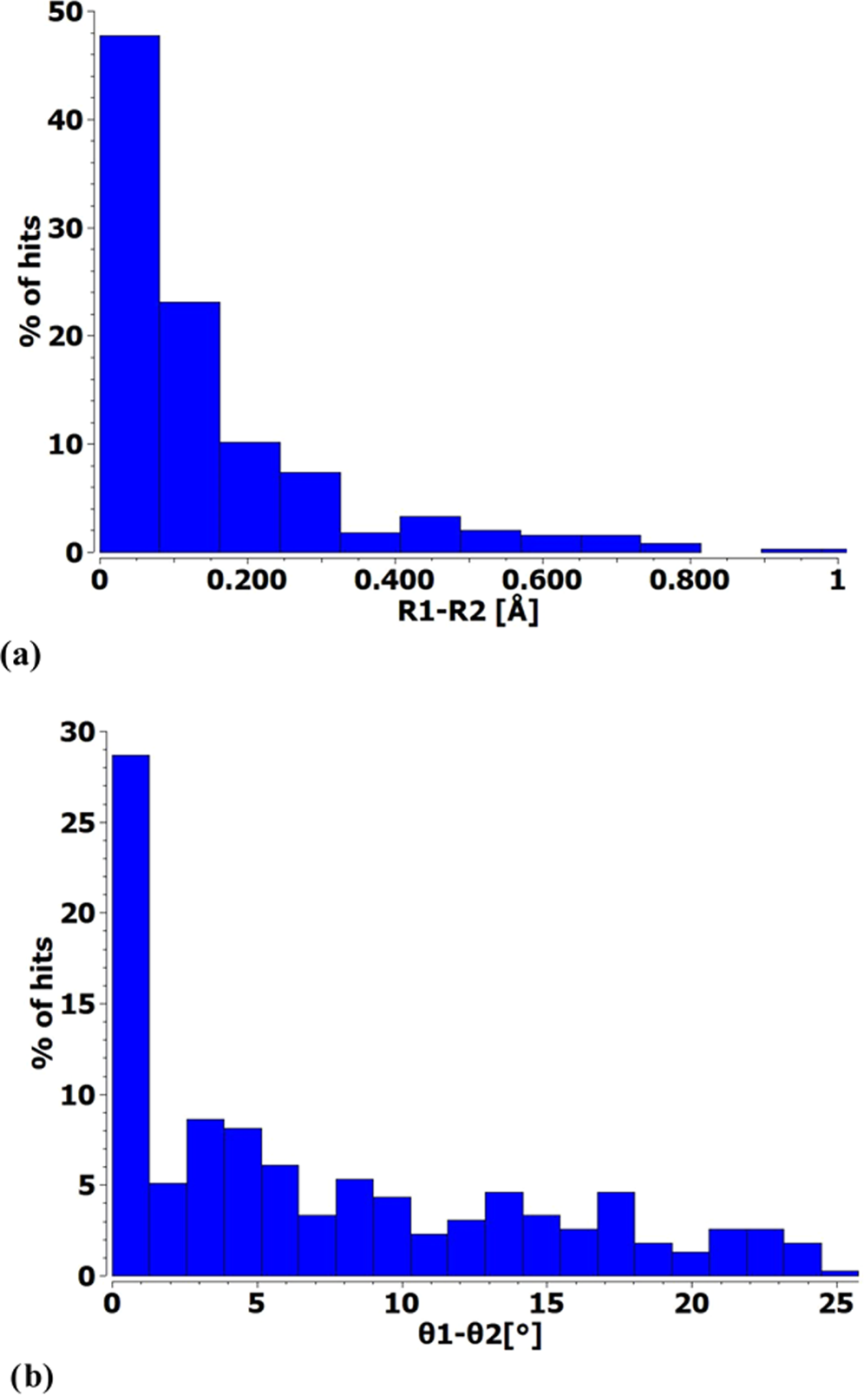
Histograms of numbers of hits of bifurcated XBs identified in CSD. The asymmetry of each arrangement is considered as the difference between the two (a) R(X···N/O) distances and (b) θ(RX···N/O) angles.

Of course, the two XBs in a bifurcated system will be of somewhat different geometry, with differing XB lengths and angles. So in addition to discussing the averages of the two XBs, it is interesting to consider the degree of asymmetry on a statistical level. Figure 4a indicates there is a surprisingly high level of symmetry regarding the two XB lengths, with most of these lengths differing by only 0.05 Å and the vast majority within 0.2 Å of one another. This quasi-symmetry extends to the two XB angles, which tend strongly toward a very small difference of only a few degrees. These trends present a picture of a generic bifurcated XB that resembles Scheme 1b, where the two LBs are placed roughly the same distance from the X atom and with comparable θ(RX···LB) angles. A heat plot that combines these two types of asymmetry is presented in Figure S2. The near symmetry of many of these bifurcated systems is reflected in several selected structures taken from the CSD and presented in Figure S4.

While there have been several prior crystal database surveys concerning the halogen bond, the current analysis presented above is the first to explicitly consider the category of bifurcated XBs. Pina *et al*. concerned themselves^66^ with decahalo-closo-carboranes (X = Cl, Br, I) and found six structures fitting their criteria of “like-like” dihalogen interactions of the B-X···X-B variety. In their comprehensive CSD search of σ-hole interactions,^67^ Frontera and Bauza looked at halogen bonding by different types of Lewis bases. The electron donor, i.e., lone pair or π-electron system, is quite common, but they also identified a number of less orthodox bases such as metal atoms donating density from their *d_z_*(2) (Ni, Pd, Rh, Pt) or *d_x^2^_*_–*y*^2^_ (Au, Ag) orbitals. The intermolecular contact distances in these latter XBs were shorter than the vdW radius sum by 0.50–0.87 Å, quite within the range described here in Figure 1; their deviation from linearity fell in the 14–37° range. A more extensive list of 31 systems was observed by Grabowski,^68^ wherein the electron donor comprises a σ-bond, in particular, cyclopropane, 1,3-cyclobutene, and cyclopentane.

Another study detailed the X···X motif associated with the porphyrin skeleton,^69^ which is quite common, with 144 examples in the CSD. As is commonly found in these dihalogen arrangements, both Type I and II were observed. Other XBs in this subgroup made use of O, N, or S as an electron donor. With some relevance to our own survey, 48% involved Br atom as σ-hole donor, I was involved in 35%, and the remaining 17% included Cl. A variation on the Lewis base theme by Hong et al.^70^ focused on the crystal structures containing Group 15 and 16 metalloids, such as As and Se. Within this grouping, S was the most common donor atom. The analysis noted that approximately 70% of X···Se interactions were close to linearity, with an average angle of 160°. Another study concerned itself chiefly with substituent and transition metal effects on halogen bonding.^71^ 289 crystal structures containing C–X···NCC and 43 synthons with the C–X···NCM motif were extracted (M = transition metal, X = Cl, Br, I). Considering a subset of this group, the X···N contact distances were shorter than the vdW sum by about 0.5 Å, roughly in the middle of our histogram in Figure 1a; the XB angles were less than 13°. As a closing remark, the F atom is widely excluded from CSD surveys dedicated to the XB due to its questionable role in this noncovalent bond.

### Quantum Chemical Probe

The geometries of the XBs contained within crystals are subject to packing forces which can easily push them away from their preferred structures in the absence of such forces. It is gratifying to see that the intrinsic preference for XB linearity is supported by the bulk of crystal structures, as delineated above. As further verification, a number of complexes were optimized, wherein a single XB is expected. F_3_CX was taken as the Lewis acid, in which the three F atoms ought to generate a sufficiently deep σ-hole on the X atom, whether Cl, Br, or I. Benzene-1,4-diol and 1,4-pyrimidine were taken as the O and N bases, respectively. These two molecules mimic the structures identified in the CSD survey in that there are two O or N atoms integrated with aromatic rings.

**Figure 5:**
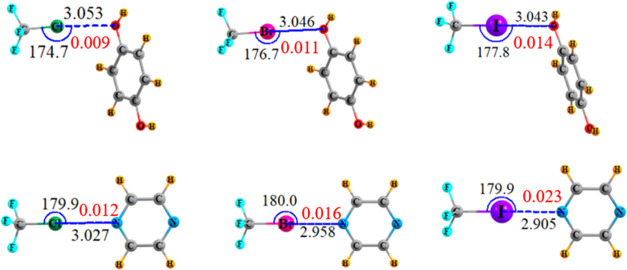
Optimized geometries of idealized monofurcated XBs. Distances in Å, angles in degs, and BCP density in red, au.

The optimized geometries are displayed in Figure 5, which verify the tendency toward linearity, particularly for the N base. Despite the larger size of the X atom in the Cl, Br, I sequence, the intermolecular distance diminishes in this same order, consistent with a stronger XB for the larger X atoms. This energetic order is confirmed by the last column of Table 3, which also suggests the N base is a more effective electron donor than is O. The red numbers in Figure 5 indicate the density at each XB bond critical point, again pointing toward increasing Cl < Br < I bond strength, and O < N.

**Table 3:** Halogen Bond Lengths and Angles of Dyads in Figure 5 and Their Counterpoise-Corrected Interaction Energies

	R(X···O/N) (Å)	θ(RX···O/N) (deg)	–*E*_int_ (kcal/mol)
Cl···O	3.053	174.7	2.17
Br···O	3.046	176.7	2.77
I···O	3.043	177.8	3.86
Cl···N	3.027	179.9	2.61
Br···N	2.958	180.0	3.79
I···N	2.905	179.9	6.04

**Figure 6:**
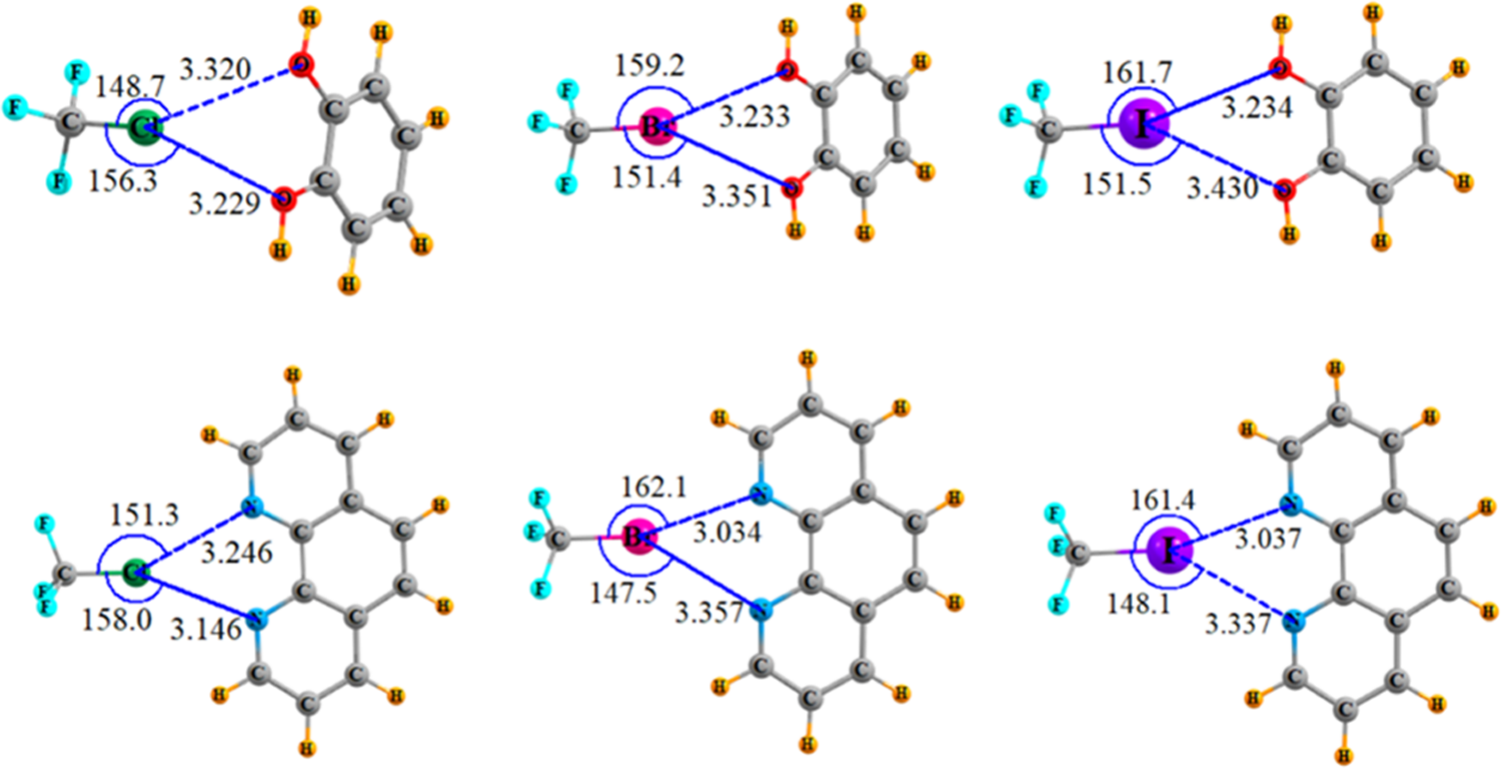
Optimized geometries of bifurcated XBs. Distances in Å, angles in degs.

With regard to bifurcated XBs, the innate preferences were assessed by placing each of the Lewis acids together with a base that contains either a pair of equally accessible O or N atoms on benzene-1,2-diol and 1,10-phenanthroline. The full geometry optimization could in principle lead to a fully symmetric bifurcated system, or a single XB involving only one of the base atoms, or something in between. What was found, however, was a preference in each case for a symmetric system, but not perfectly so, as may be seen in Figure 6. The average of the two XB lengths listed in Table 4 are all a bit longer than the same lengths in the monofurcated dimers, and there is little dependence on the nature of the X atom. These longer bifurcated XBs are consistent with the CSD trends enumerated above. The X···N distances tend to be a bit shorter than X···O but only by a small margin. The next column of Table 4 shows that the two XB lengths in a bifurcated XB differ from one another by a variable amount. There is an increasing asymmetry as the X atom grows in size, and this asymmetry is larger for N than for O. The magnitude of R_1_–R_2_ varies between 0.09 and 0.32 Å, a range which fits nicely with the length asymmetries of crystals denoted in Figure 4a.

**Table 4:** Halogen Bond Lengths and Angles of Triads in Figure 6 and Their Counterpoise-Corrected Interaction Energies

	*R*_mean_ (Å)	R_1_–R_2_ (Å)	θ_mean_ (deg)	θ_1_–θ_2_ (deg)	–*E*_int_ (kcal/mol)
Cl···O/O	3.275	0.091	152.5	7.6	2.52
Br···O/O	3.292	0.118	155.3	7.8	3.22
I···O/O	3.332	0.196	156.6	10.2	4.52
Cl···N/N	3.196	0.100	154.7	6.7	4.50
Br···N/N	3.196	0.323	154.8	14.6	6.11
I···N/N	3.187	0.300	154.8	13.3	9.16

Of course, both of the two XBs must deviate from linearity in order for both to be present. The mean θ angle in Table 4 tends toward 155°, with little sensitivity to either X or base atom. This 25° nonlinearity fits nicely within the scope of angles observed within a full set of crystals in Figure 3b. Rather than a precisely symmetric arrangement with both θ(RX···N/O) angles equal to one another, the optimized difference between these two angles varies between 7 and 15°, listed in the penultimate column of Table 4. This range fits nicely into the set of crystal angular asymmetries illustrated in Figure 4b.

The last column of Table 4 contains the interaction energy of the entire trimer. Just as for the monofurcated systems, this quantity rises along with the size of the X atom. However, the interaction energies of the N/N triads are roughly double those of the O/O analogues, a distinction not observed in the dyads. The trimer energies also suffer from the negative cooperativity anticipated when the X atom serves as a double electron acceptor. The interaction energies of the triads in Table 4 are far less than double the quantities in Table 3. These reduced quantities can also be attributed to the angular deformations of the two XBs in the triad, as compared to the linear single XBs in the dimers. The full AIM molecular diagrams of the various bifurcated systems are contained in Figure S5.

The information presented here can be placed in the context of some earlier studies of bifurcated halogen-bonded systems. Novák et al.^44^ examined three dihalogen molecules, ClF, BrF, and BrCl, in complexes with a variety of electron donor sites of 4-substituted 1,2-dimethoxybenzenes, both charged and neutral, where the bifurcated X···O/O halogen bond motif exists. The M06-2X interaction energies for complexes with neutral Lewis bases (as the benzene-1,2-diol in the current work) lie between −5.0 and −9.0 kcal/mol, more strongly bound than our related bifurcated model where the interaction energy is −3.2 kcal/mol. Complexes with substituents -CH_3_ or -CF_3_ were more tightly bound as their electron-donating ability enhanced the nucleophilicity of the Lewis base. Within the wide group of 38 neutral complexes therein, the interatomic Br···O distances in the range between 2.614 and 3.287 Å are generally shorter than those obtained in the current work, in line with their greater stability.

Substituent and cooperative effects of symmetric bifurcated halogen bonds were studied by Esrafili et al.^43^ when NCX (X = Cl, Br) was paired with a series of N-formyl formamide derivatives. Among the group of binary complexes stabilized by Cl or Br···O-bifurcated halogen bonds, MP2 interaction energies of −3.3 to −7.2 kcal/mol were measured for Cl dyads and in the range between −3.9 and −8.5 kcal/mol for Br. Our Cl and Br complexes with O-electron donors are slightly weaker, 2.5–3.2 kcal/mol. Their systems were designed as examples of perfectly symmetrical bifurcation of halogen bonds, with equal X···O=C angles of 152–153°, similar to the arithmetic mean of the cases examined here. Their halogen bond distances were shorter, in line with their larger interaction energy. Despite these differences, the BCP electron densities were in line with our data. For the strongest complexes involving Li, this density was 0.009 and 0.011 au for Cl and Br adducts, respectively, quite similar to the values extracted in our models.

Bifurcated X···O interactions were also inspected by de Oliveira et al.,^45^ who considered CFCl_3_···ozone complexes. The Cl···O distances were similar but not identical: 3.414 and 3.431 Å. This complexation with O_3_ is less stable than the one with benzene-1,2-diol considered here. Indeed, the MP2 interaction energies amounted to only 0.4 kcal/mol, with the BCP ρ of 0.004 au.

These prior publications concerning bifurcated halogen bonds were limited to only Cl···O and Br···O combinations. The work described here extends this listing to those involving I, as well as adding N to the grouping of electron donor atoms. Perhaps more importantly, a detailed statistical analysis of real structures from CSD has been carried out to validate some of the trends observed from the calculations.

It should be noted parenthetically that the bifurcated interaction term can have an alternate meaning. In addition to the idea presented here that the electron acceptor is associated with a pair of bases, a converse geometry places two Lewis acids in position to accept electron density from a single nucleophilic lone pair.^15,72^ This particular arrangement has seen some computational studies from the perspective of halogen bonds and how they compare with H-bonds ^21^. It was found, for example, that the latter configuration can be energetically preferred in certain situations.

## Conclusions

Based on calculations of model systems, there is a marked propensity for an XB to be linear and with a R(X···O/N) bond length of about 3.0 Å. This distance elongates by about 0.2–0.3 Å when a second base is added, and the two electron donors are situated about 155° from the R–X bond. The optimal location of the two bases in the bifurcated arrangement is somewhat displaced from symmetry. The two XB lengths differ by some 0.1–0.3 Å, and the two θ(RX···O/N) angles by 7–15°. The asymmetry of these two bonds is most noticeable for the heavier X atom and more so for N than for O. Due to the nonlinearity and negative cooperativity, the presence of the second XB offers only a small increment in the interaction energy.

These trends are largely reproduced within a crystal environment, although with occasional large deviations caused by attendant packing forces. In the first place, the preference for linearity is present, particularly when cone correction is applied. XB lengths vary a great deal, and the contraction versus the vdW sum is quite obvious, with many contracted by as much as 0.5 Å. The predicted reduction of the XB angle in the bifurcated systems is clearly present, with a peak that nearly matches the predicted mean value of 155°. Also reproduced is the elongation of the XB lengths in the bifurcated situation. The model systems tend toward a certain degree of asymmetry in the two XBs. Although there are quite a few crystal systems with a small difference in the two angles, there are also many with a larger discrepancy, some as large as 25°. The observed XB bond length difference tends to be less than about 0.3 Å, nicely matching the quantum calculations of the idealized systems.

Finally, the transition from mono to bifurcated XB arrangements perturbs the propensity of individual halogen atoms to participate in XBs. The proportion of Br···O XBs is lowered, and there is a shift away from Cl···O to Cl···N. Most dramatic is the increase in I XBs, which rise from 21% of all monofurcated to half of all bifurcated arrangements. Overall, although quite a number of bifurcated XBs occur within crystals, they are relatively rare, accounting for less than 2% of standard single XBs.

## Supporting Information Available

Percentages of different sorts of monofurcated XBs (a) and bifurcated XBs (b) identified in CSD (Figure S1); heat plot representing the R-X···LB average angle versus the mean X···LB distance deviation for bifurcated halogen bond geometries (Figure S2); heat plot of asymmetric factors of structures from the CSD (Figure S3); AIM molecular diagrams of selected structures from the CSD (Figure S4); AIM molecular diagrams of fully optimized complexes containing mono and bifurcated XBs. The density of each bond critical point is displayed in au (Figure S5); crystal structures coordinates (PDF)
