# Supplementary material for: Crystal Structure Survey and Theoretical Analysis of Bifurcated Halogen Bonds
Source: Cryst Growth Des. 2022 Oct 10;22(11):6521–30. doi: 10.1021/acs.cgd.2c00726 (PMC9634799; doi:10.1021/acs.cgd.2c00726)
Supplement: Supplementary file 1 — cg2c00726_si_001.pdf [file cg2c00726_si_001.pdf]

Crystal Structure Survey and Theoretical Analysis of Bifurcated Halogen Bonds

Mariusz Michalczyk*[1] Wiktor Zierkiewicz[1] and Steve Scheiner[2]

[1]Faculty of Chemistry, Wrocław University of Science and Technology, Wybrzeże Wyspiańskiego 27, 50-370 Wrocław, Poland
[2]Department of Chemistry and Biochemistry, Utah State University Logan, Utah 84322-0300, United States

*Correspondence to: mariusz.michalczyk@pwr.edu.pl

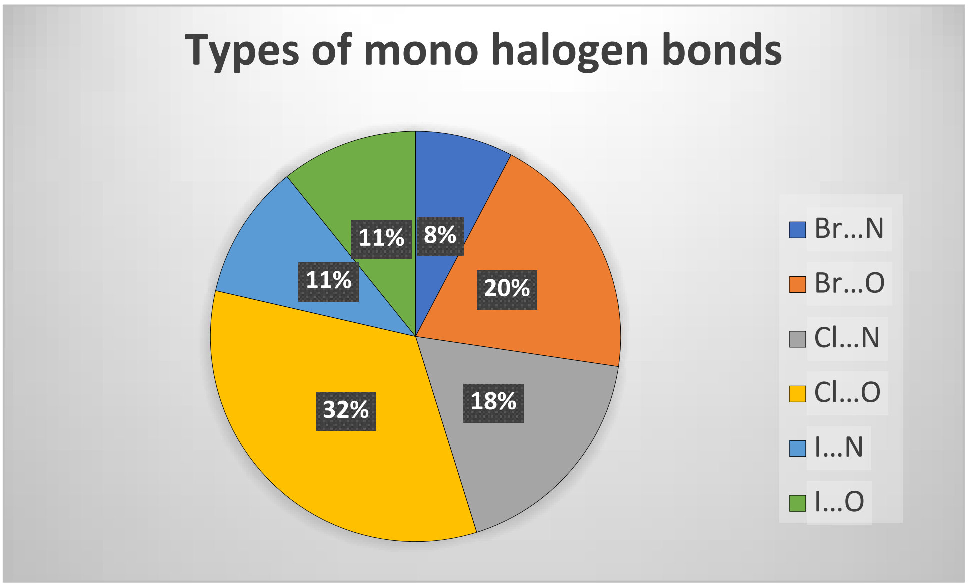

a)

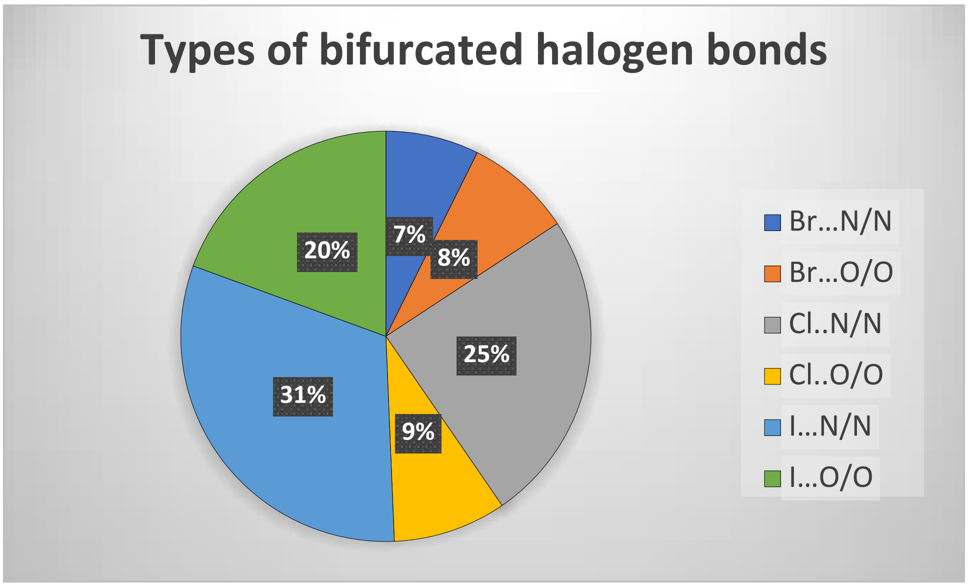

b)

Fig. S1. Percentages of different sorts of monofurcated XBs (a) and bifurcated XBs (b) identified in CSD.

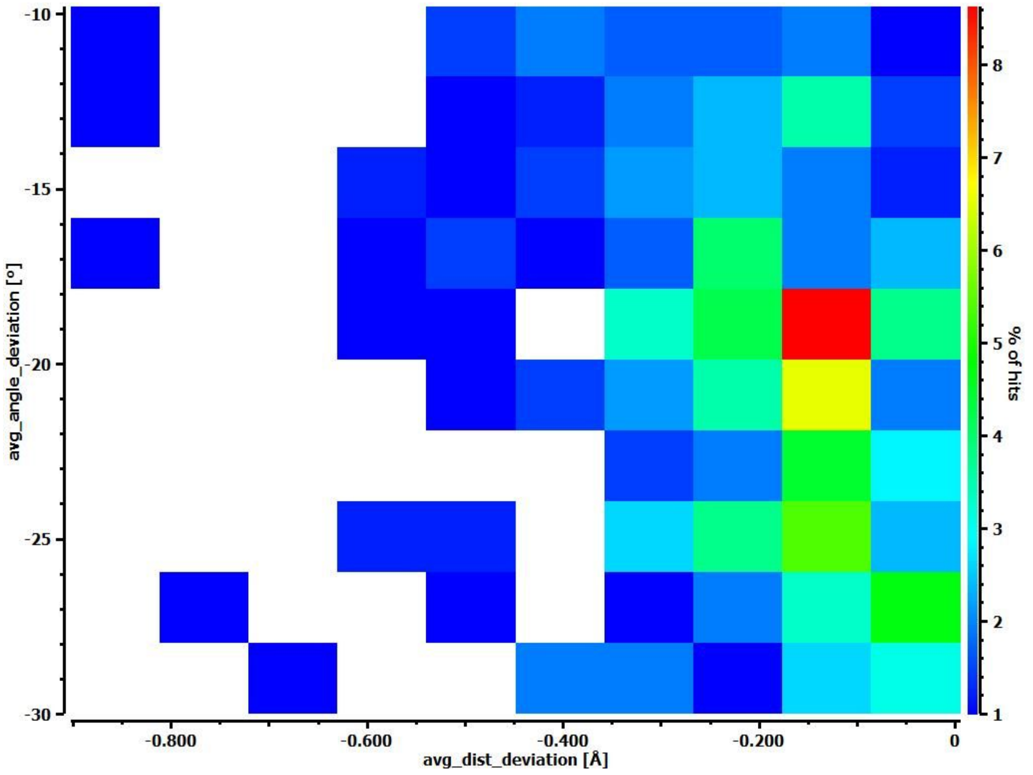

Fig. S2. Heat plot representing the R-X···LB average angle versus the mean X···LB distance deviation for bifurcated halogen bond geometries.

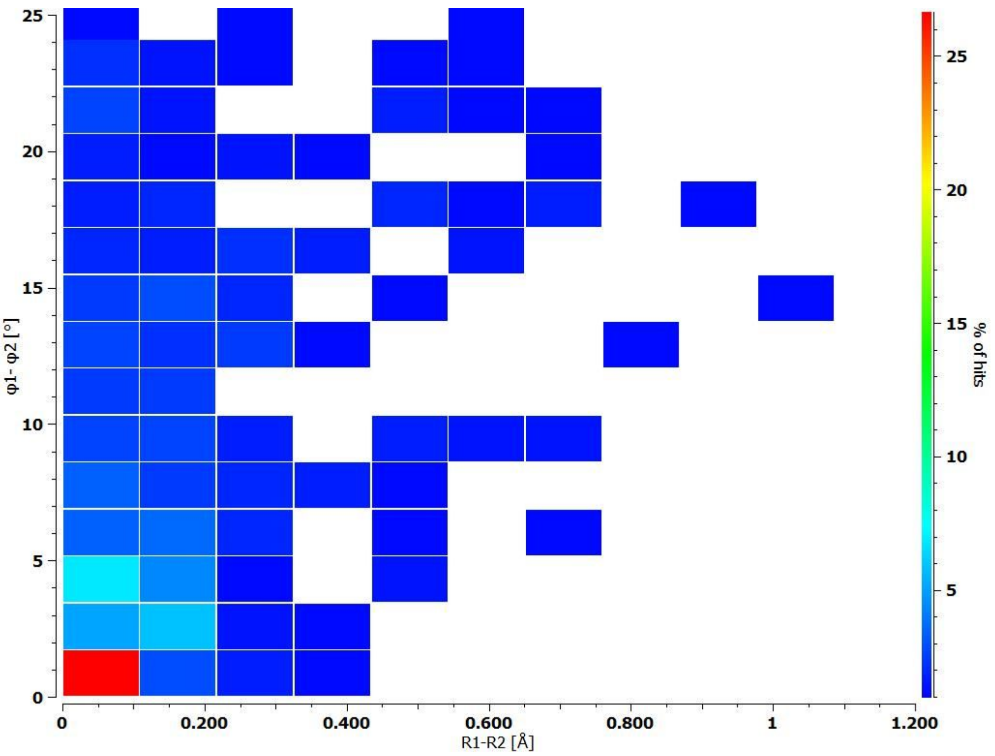

Fig S3. Heat plot of asymmetric factors of structures from the CSD.

Halogen	double O,O
Cl	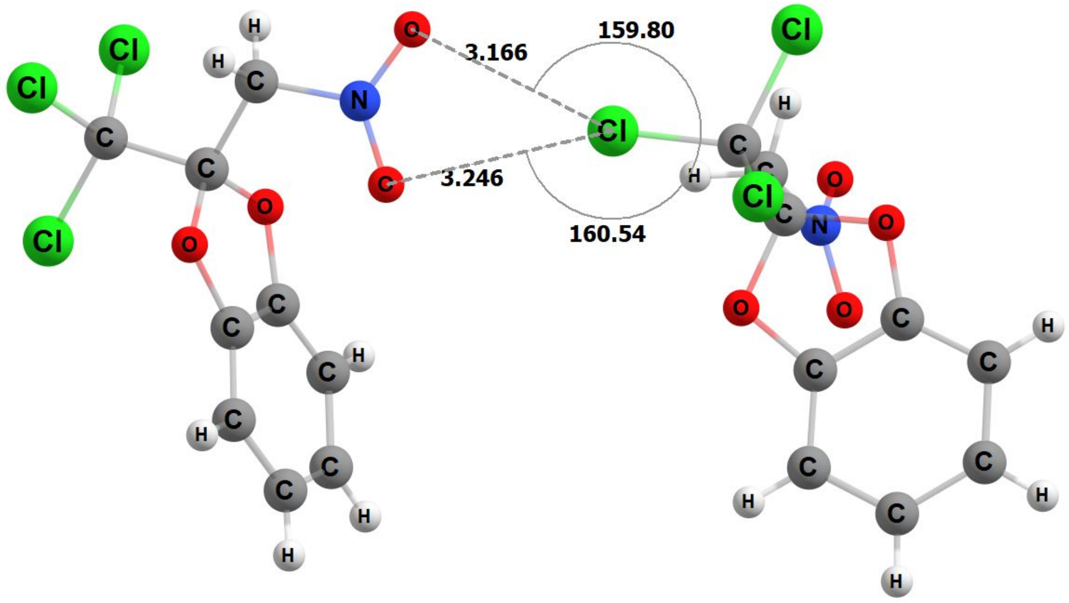 HIVHEB

Br	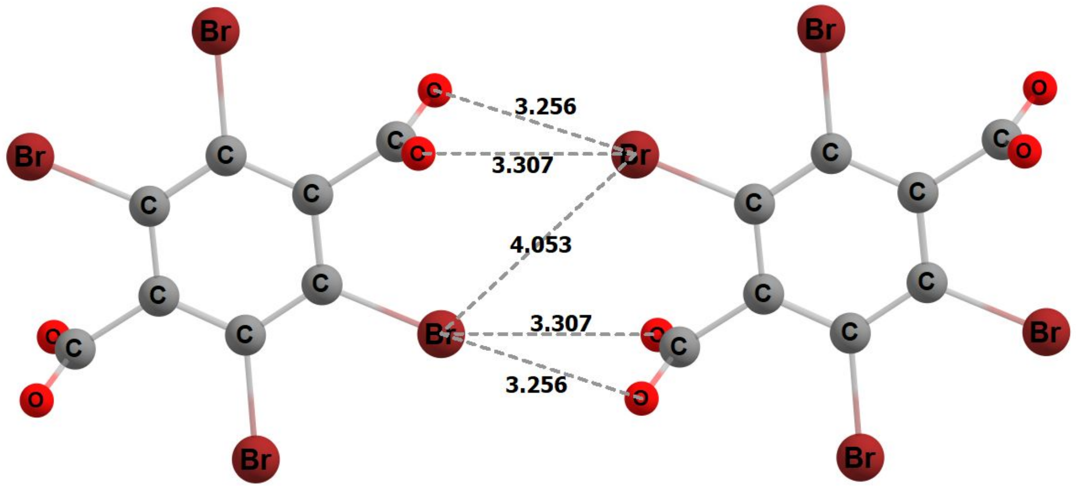 ATOVUD
I	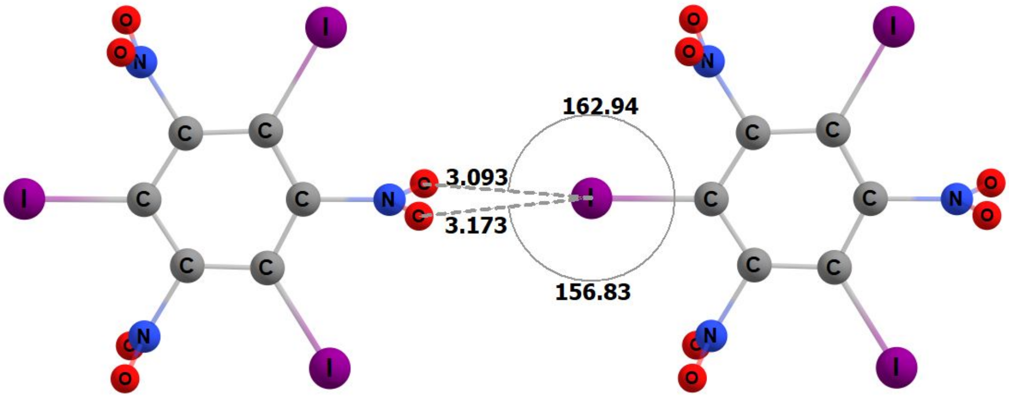 NAHTEY
	double N,N
Cl	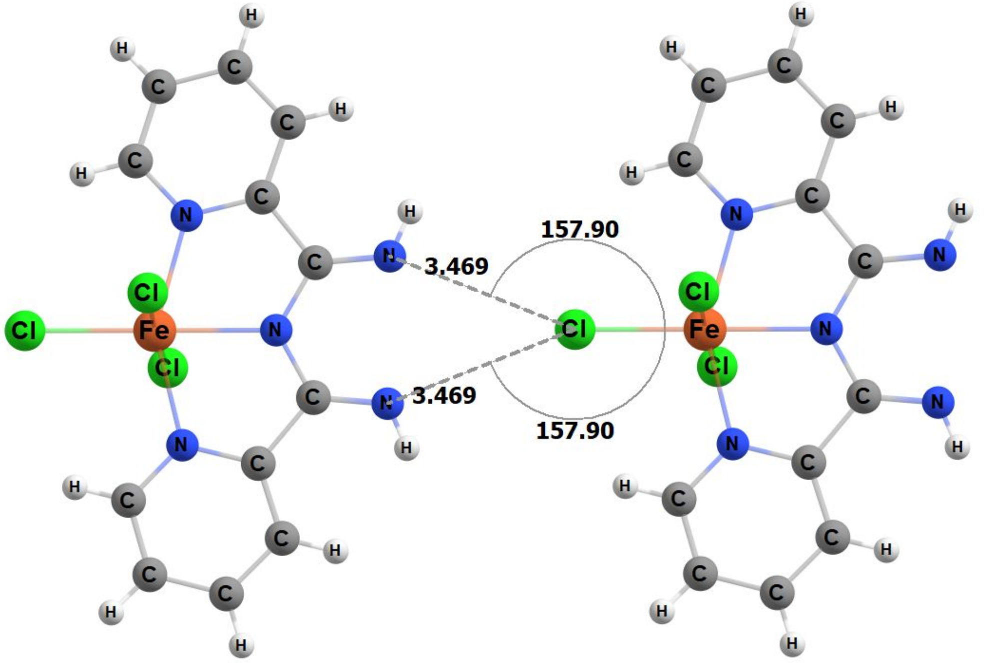 SEMREK

Br	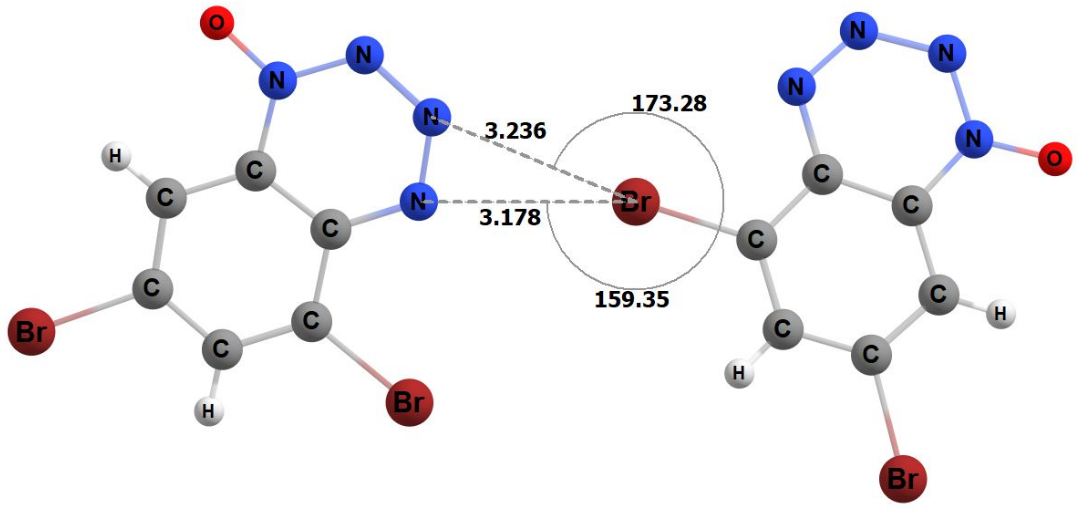 HERBAH
I	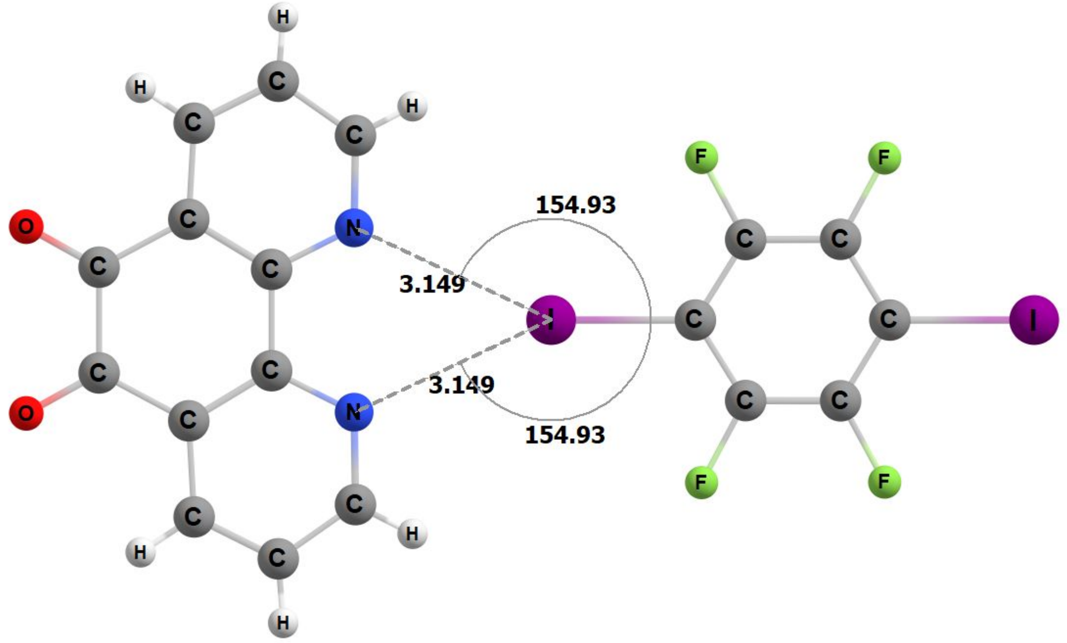 EXIFEX

Fig S4. AIM molecular diagrams of selected structures from the CSD.

Mono	
Cl…O	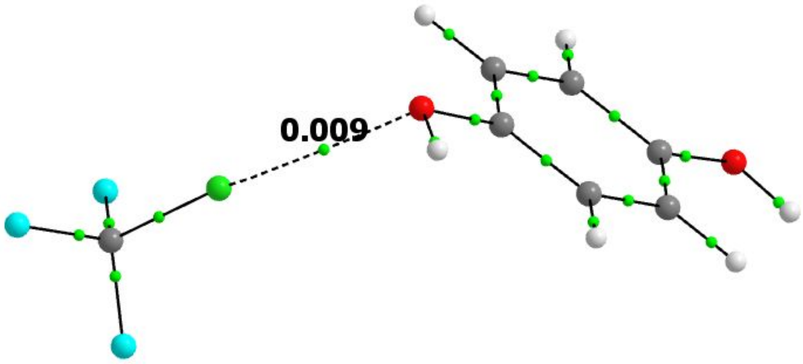
Br…O	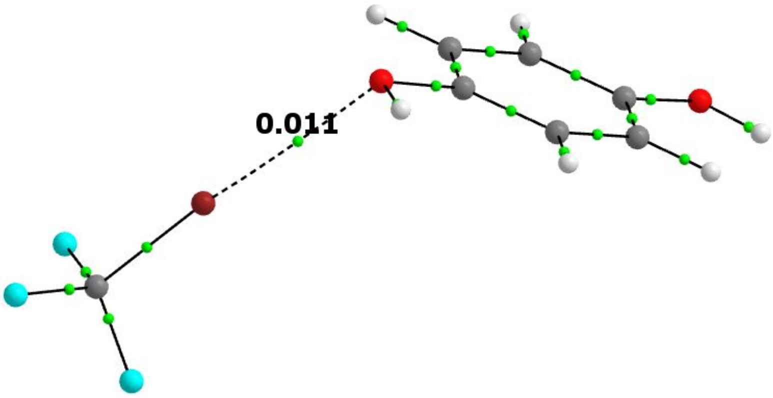
I…O	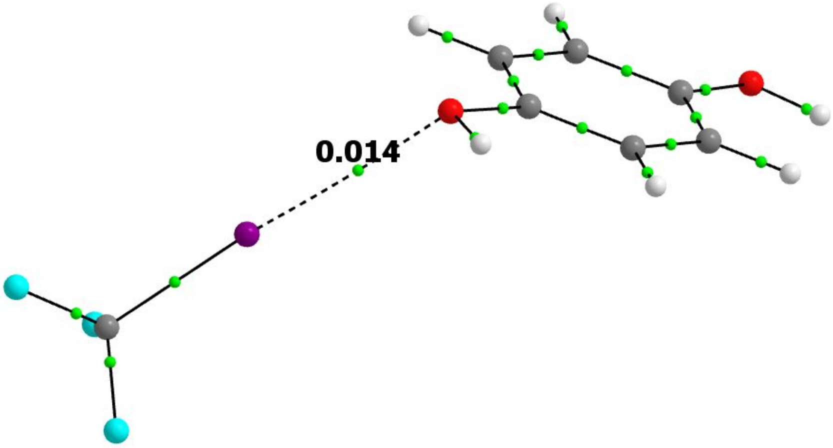
Cl…N	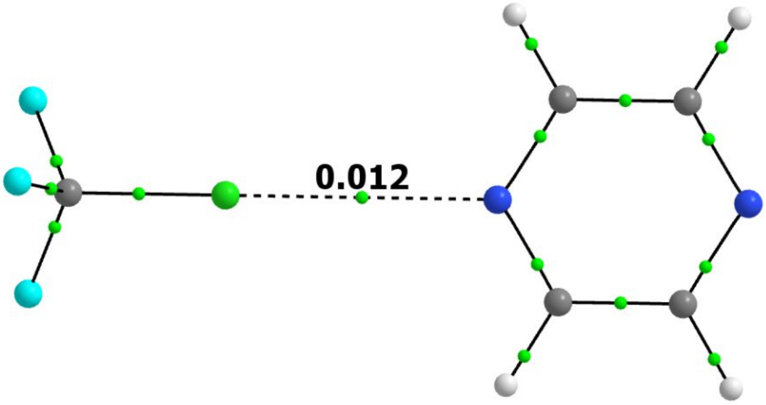

Br…N	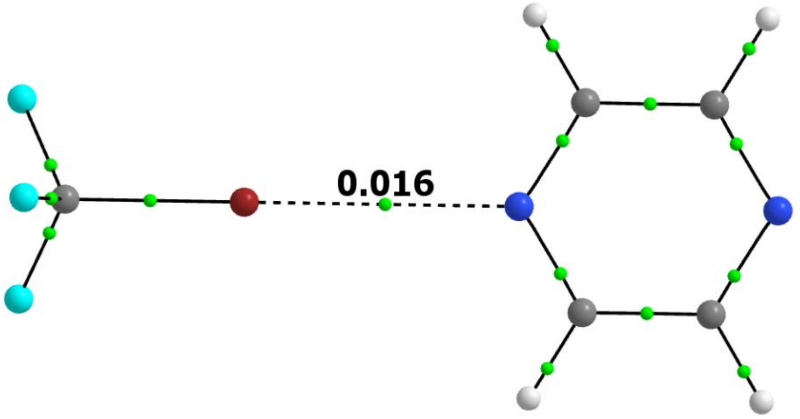
I…N	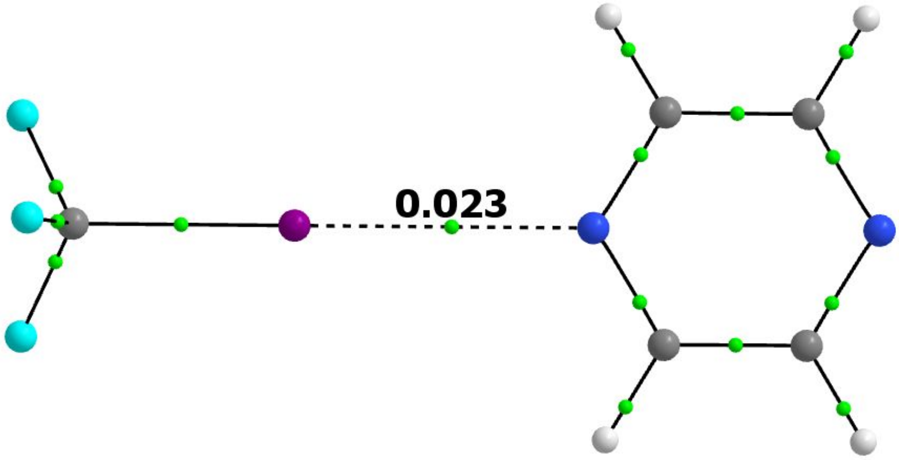

Bifurcated	
Cl…O/O	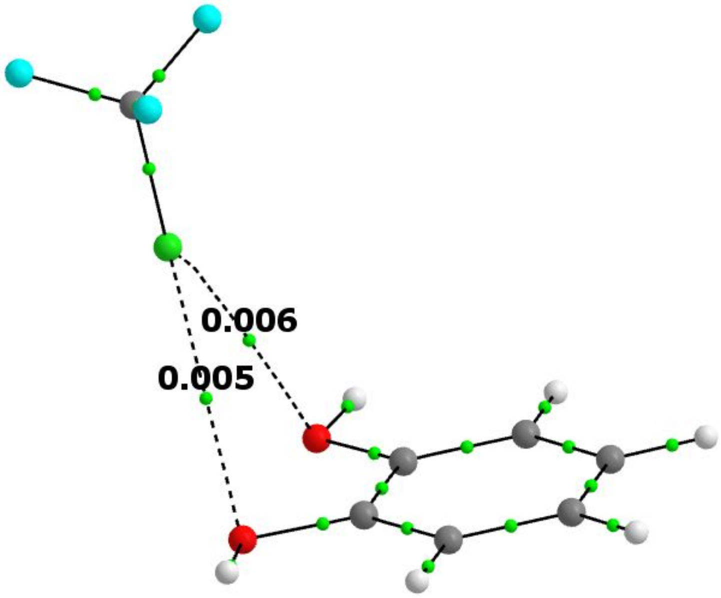

Br…O/O	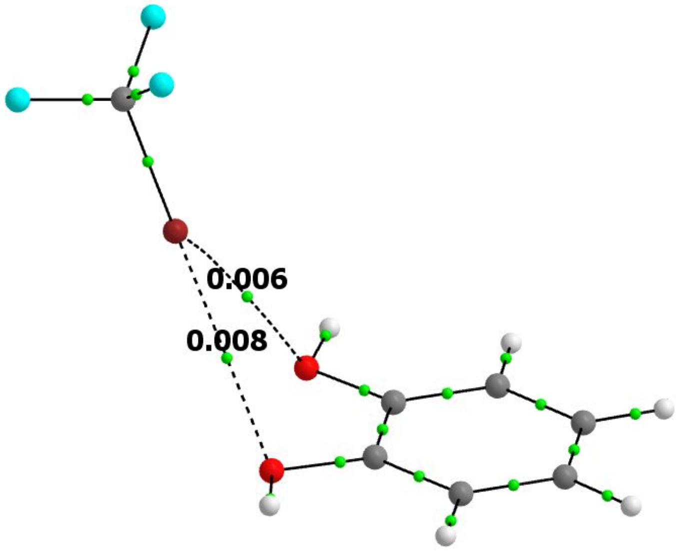
I…O/O	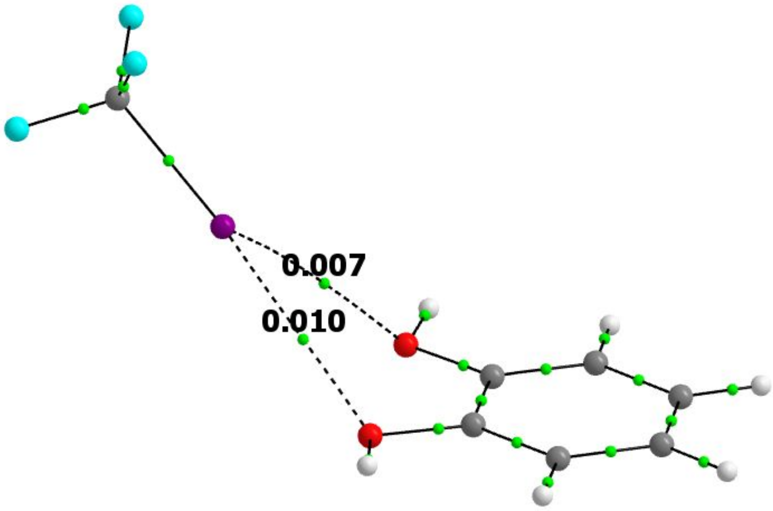
Cl…N/N	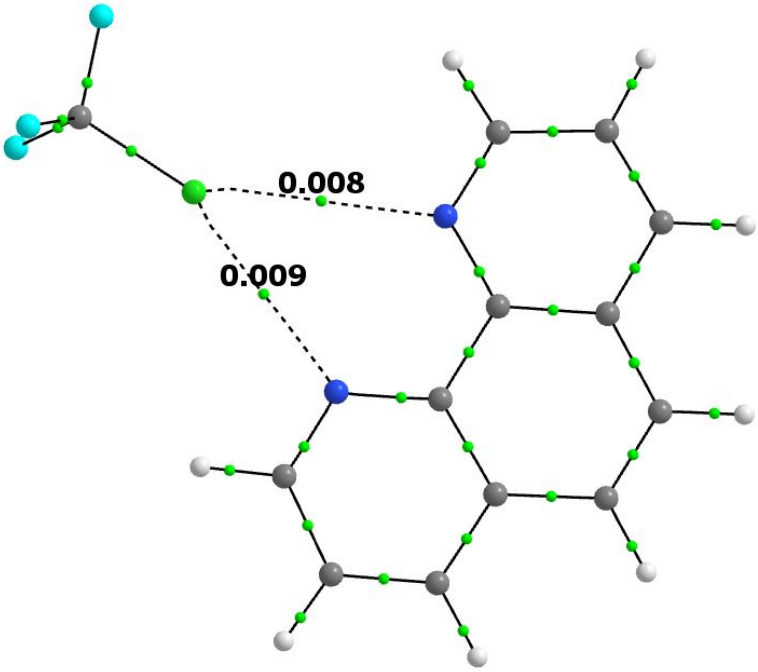

Br…N/N	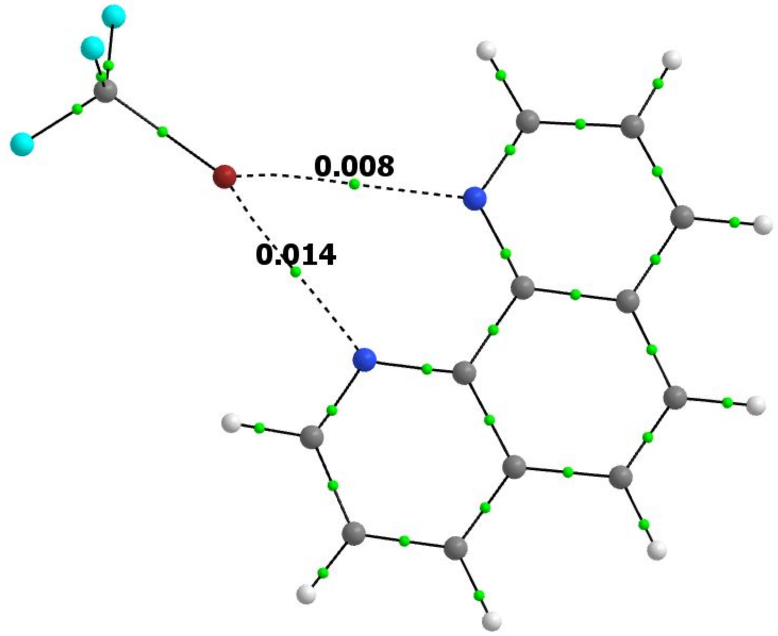
I…N/N	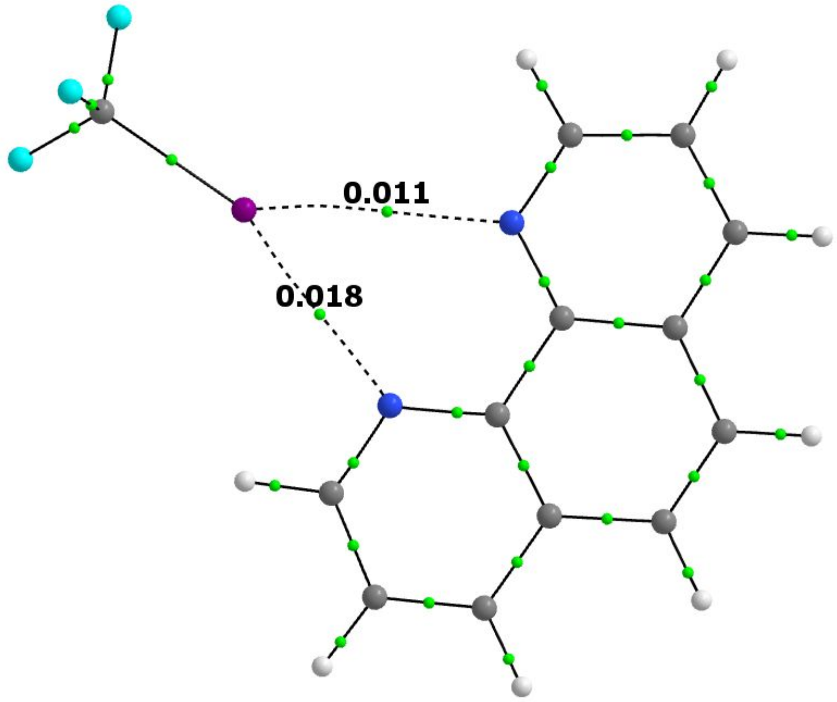

Fig S5. AIM molecular diagrams of fully optimized complexes containing mono and bifurcated XBs. The density of each bond critical point is displayed in au.

Coordinates

Crystal structures

Halogen	double O,O			
Cl	HIVHEB			
	Cl	5.08360000	6.93580000	4.71560000
	Cl	2.69540000	8.52570000	4.39370000
	Cl	5.05120000	9.15190000	2.85810000
	O	4.76370000	6.42970000	1.71540000
	O	1.18120000	4.24210000	2.36210000
	O	2.79890000	7.62940000	1.51910000
	O	2.58250000	4.84590000	0.83480000
	N	2.14260000	4.88050000	1.97440000
	C	3.61500000	6.88350000	2.41510000
	C	3.12530000	8.30680000	-0.83920000
	H	2.31560000	8.79820000	-0.91070000
	C	4.01420000	8.17420000	-1.91860000
	H	3.80000000	8.58400000	-2.74820000
	C	5.19850000	7.45760000	-1.80190000
	H	5.77580000	7.38950000	-2.55350000
	C	5.56300000	6.82930000	-0.59610000
	H	6.37120000	6.33830000	-0.50600000
	C	4.67610000	6.97040000	0.44100000
	C	3.49840000	7.68340000	0.32270000
	C	2.82790000	5.71880000	3.00900000
	H	3.44540000	5.14570000	3.52910000
	H	2.14860000	6.07700000	3.63450000
	C	4.09930000	7.84180000	3.55830000
	Cl	9.88470000	2.88800000	9.78610000
	Cl	7.49640000	1.29810000	9.46410000
	Cl	9.85230000	0.67190000	7.92860000
	O	9.56480000	3.39410000	6.78590000
	O	5.98220000	5.58170000	7.43260000
	O	7.60000000	2.19440000	6.58960000
	O	7.38360000	4.97790000	5.90530000
	N	6.94370000	4.94330000	7.04490000
	C	8.41610000	2.94030000	7.48550000
	C	7.92630000	1.51700000	4.23130000
	H	7.11660000	1.02560000	4.15980000
	C	8.81530000	1.64960000	3.15190000
	H	8.60110000	1.23980000	2.32230000
	C	9.99960000	2.36620000	3.26850000
	H	10.57690000	2.43430000	2.51700000
	C	10.36410000	2.99450000	4.47440000
	H	11.17230000	3.48550000	4.56440000
	C	9.47720000	2.85340000	5.51150000
	C	8.29940000	2.14040000	5.39320000
	C	7.62900000	4.10500000	8.07950000
	H	8.24640000	4.67810000	8.59950000
	H	6.94970000	3.74680000	8.70500000
	C	8.90030000	1.98200000	8.62870000
Br	ATOVUD			
	Br	4.85000000	4.29490000	6.95120000
	Br	7.63910000	5.62540000	5.60700000
	O	1.79530000	6.23860000	6.73010000
	O	1.76800000	4.42760000	5.42240000
	C	4.76280000	5.60640000	5.58580000
	C	5.91820000	6.11980000	5.00620000
	C	3.50960000	6.01900000	5.15020000
	C	2.23600000	5.52470000	5.83460000
	Br	4.49490000	8.76010000	2.16250000
	Br	1.70580000	7.42960000	3.50680000
	O	7.54960000	6.81640000	2.38370000
	O	7.57690000	8.62740000	3.69130000
	C	4.58210000	7.44860000	3.52790000
	C	3.42670000	6.93520000	4.10760000
	C	5.83530000	7.03590000	3.96360000
	C	7.10890000	7.53030000	3.27910000
	Br	13.74320000	4.29490000	6.95120000
	Br	16.53230000	5.62540000	5.60700000
	O	10.68850000	6.23860000	6.73010000
	O	10.66120000	4.42760000	5.42240000
	C	13.65600000	5.60640000	5.58580000

	C	14.81140000	6.11980000	5.00620000
	C	12.40280000	6.01900000	5.15020000
	C	11.12920000	5.52470000	5.83460000
	Br	13.38810000	8.76010000	2.16250000
	Br	10.59900000	7.42960000	3.50680000
	O	16.44280000	6.81640000	2.38370000
	O	16.47010000	8.62740000	3.69130000
	C	13.47530000	7.44860000	3.52790000
	C	12.31990000	6.93520000	4.10760000
	C	14.72850000	7.03590000	3.96360000
	C	16.00210000	7.53030000	3.27910000
**I**	NAHTEY			
	I	2.98090000	14.75590000	3.19810000
	I	-0.91790000	17.80800000	6.72660000
	O	3.57020000	17.80800000	1.40090000
	O	4.99120000	17.80800000	3.02290000
	O	1.13230000	14.88870000	6.39300000
	O	-0.30330000	14.88750000	4.77100000
	N	3.85610000	17.80800000	2.57090000
	N	0.63920000	15.35050000	5.38970000
	C	2.24630000	16.57880000	3.96530000
	C	2.71750000	17.80800000	3.54220000
	C	1.21400000	16.62320000	4.89330000
	C	0.67020000	17.80800000	5.38520000
	I	2.98090000	20.86010000	3.19810000
	O	1.13230000	20.72730000	6.39300000
	O	-0.30330000	20.72850000	4.77100000
	N	0.63920000	20.26550000	5.38970000
	C	2.24630000	19.03720000	3.96530000
	C	1.21400000	18.99280000	4.89330000
	I	-4.35140000	14.75590000	9.73830000
	I	-8.25020000	17.80800000	13.26670000
	O	-3.76210000	17.80800000	7.94110000
	O	-2.34110000	17.80800000	9.56300000
	O	-6.20000000	14.88870000	12.93320000
	O	-7.63560000	14.88750000	11.31120000
	N	-3.47620000	17.80800000	9.11110000
	N	-6.69310000	15.35050000	11.92990000
	C	-5.08600000	16.57880000	10.50550000
	C	-4.61480000	17.80800000	10.08230000
	C	-6.11830000	16.62320000	11.43350000
	C	-6.66210000	17.80800000	11.92530000
	I	-4.35140000	20.86010000	9.73830000
	O	-6.20000000	20.72730000	12.93320000
	O	-7.63560000	20.72850000	11.31120000
	N	-6.69310000	20.26550000	11.92990000
	C	-5.08600000	19.03720000	10.50550000
	C	-6.11830000	18.99280000	11.43350000
	**double N,N**			
**Cl**	SEMREK			
	Fe	7.38510000	1.87690000	2.59830000
	Cl	5.32380000	1.92910000	3.83320000
	Cl	7.38510000	-0.36280000	2.59830000
	N	7.38510000	3.97260000	2.59830000
	N	8.42830000	2.39150000	4.36940000
	N	8.09560000	5.91330000	3.69280000
	H	8.54560000	6.25400000	4.33400000
	C	8.66610000	3.69830000	4.57930000
	C	8.04200000	4.63120000	3.59300000
	C	8.88800000	1.48430000	5.26010000
	H	8.70980000	0.56140000	5.11930000
	C	9.42330000	4.12630000	5.65400000
	H	9.62340000	5.04790000	5.76970000
	C	9.60510000	1.87150000	6.36380000
	H	9.90550000	1.22420000	6.99070000
	C	9.88180000	3.18870000	6.55510000
	H	10.39170000	3.46230000	7.30830000
	N	6.34190000	2.39150000	0.82730000
	N	6.67460000	5.91330000	1.50390000
	H	6.22460000	6.25400000	0.86260000
	C	6.10400000	3.69830000	0.61740000
	C	6.72820000	4.63120000	1.60370000
	C	5.88220000	1.48430000	-0.06340000
	H	6.06040000	0.56140000	0.07740000
	C	5.34690000	4.12630000	-0.45730000

	H	5.14670000	5.04790000	-0.57300000
	C	5.16510000	1.87150000	-1.16720000
	H	4.86470000	1.22420000	-1.79400000
	C	4.88840000	3.18870000	-1.35840000
	H	4.37850000	3.46230000	-2.11160000
	Cl	9.44640000	1.92910000	1.36350000
	Fe	7.38510000	-7.61330000	2.59830000
	Cl	5.32380000	-7.56110000	3.83320000
	Cl	7.38510000	-9.85300000	2.59830000
	N	7.38510000	-5.51760000	2.59830000
	N	8.42830000	-7.09870000	4.36940000
	N	8.09560000	-3.57690000	3.69280000
	H	8.54560000	-3.23620000	4.33400000
	C	8.66610000	-5.79190000	4.57930000
	C	8.04200000	-4.85900000	3.59300000
	C	8.88800000	-8.00590000	5.26010000
	H	8.70980000	-8.92880000	5.11930000
	C	9.42330000	-5.36390000	5.65400000
	H	9.62340000	-4.44230000	5.76970000
	C	9.60510000	-7.61870000	6.36380000
	H	9.90550000	-8.26600000	6.99070000
	C	9.88180000	-6.30150000	6.55510000
	H	10.39170000	-6.02790000	7.30830000
	N	6.34190000	-7.09870000	0.82730000
	N	6.67460000	-3.57690000	1.50390000
	H	6.22460000	-3.23620000	0.86260000
	C	6.10400000	-5.79190000	0.61740000
	C	6.72820000	-4.85900000	1.60370000
	C	5.88220000	-8.00590000	-0.06340000
	H	6.06040000	-8.92880000	0.07740000
	C	5.34690000	-5.36390000	-0.45730000
	H	5.14670000	-4.44230000	-0.57300000
	C	5.16510000	-7.61870000	-1.16720000
	H	4.86470000	-8.26600000	-1.79400000
	C	4.88840000	-6.30150000	-1.35840000
	H	4.37850000	-6.02790000	-2.11160000
	Cl	9.44640000	-7.56110000	1.36350000
**Br**	HERBAH			
	Br	4.26790000	-0.80320000	4.93760000
	Br	0.09360000	-2.51690000	8.30310000
	O	1.17540000	2.70600000	9.25730000
	N	1.97740000	2.39150000	8.36760000
	N	2.85330000	3.31600000	7.95500000
	N	3.75720000	2.98910000	7.01050000
	N	3.83470000	1.82350000	6.43950000
	C	3.01090000	-0.44740000	6.28300000
	C	2.14390000	-1.42230000	6.73470000
	C	1.26280000	-1.13180000	7.73990000
	C	1.12810000	0.12220000	8.29910000
	C	2.95570000	0.86430000	6.84620000
	C	1.98780000	1.08880000	7.83370000
	H	2.16990000	-2.30230000	6.35150000
	H	0.47330000	0.31040000	8.97570000
	Br	6.13410000	-4.93070000	4.83990000
	Br	10.30840000	-6.64440000	1.47440000
	O	9.22660000	-1.42150000	0.52020000
	N	8.42460000	-1.73600000	1.40990000
	N	7.54870000	-0.81150000	1.82250000
	N	6.64480000	-1.13840000	2.76700000
	N	6.56730000	-2.30400000	3.33800000
	C	7.39110000	-4.57490000	3.49450000
	C	8.25810000	-5.54980000	3.04280000
	C	9.13920000	-5.25930000	2.03760000
	C	9.27390000	-4.00530000	1.47840000
	C	7.44630000	-3.26320000	2.93130000
	C	8.41420000	-3.03870000	1.94380000
	H	8.23210000	-6.42980000	3.42600000
	H	9.92870000	-3.81710000	0.80180000
**I**	EXIFEX			
	C	0.73370000	3.83020000	2.43930000
	C	1.24230000	4.99770000	1.93960000
	C	2.22290000	4.99460000	0.97620000
	C	2.74420000	3.83020000	0.46940000
	F	0.78560000	6.17360000	2.38560000
	F	2.67800000	6.18130000	0.53010000

	I -0.80150000 3.83030000 3.85520000
	I 4.27290000 3.83020000 -0.95160000
	C 1.24230000 2.66280000 1.93960000
	C 2.22290000 2.66590000 0.97620000
	F 0.78560000 1.48690000 2.38560000
	F 2.67800000 1.47920000 0.53010000
	C 7.24930000 4.56260000 -3.69230000
	C 8.20250000 5.28730000 -4.42560000
	C 8.15240000 6.67690000 -4.36490000
	H 8.76580000 7.18860000 -4.84130000
	C 7.19950000 7.28360000 -3.60350000
	H 7.15300000 8.21050000 -3.54980000
	C 6.31210000 6.49920000 -2.91810000
	H 5.66290000 6.92200000 -2.40430000
	C 9.22460000 4.59480000 -5.23010000
	N 6.31860000 5.16320000 -2.94030000
	O 10.04460000 5.18000000 -5.89460000
	C 7.24930000 3.09790000 -3.69230000
	C 8.20250000 2.37320000 -4.42560000
	C 8.15240000 0.98360000 -4.36490000
	H 8.76580000 0.47190000 -4.84130000
	C 7.19950000 0.37690000 -3.60350000
	H 7.15300000 -0.55000000 -3.54980000
	C 6.31210000 1.16130000 -2.91810000
	H 5.66290000 0.73850000 -2.40430000
	C 9.22460000 3.06570000 -5.23010000
	N 6.31860000 2.49730000 -2.94030000
	O 10.04460000 2.48050000 -5.89460000

Theoretical models

Monohalogen	
Cl…O	C -3.09416200 -1.46504500 -4.99918300
	F -3.81439800 -0.79651600 -5.88665700
	F -2.13125000 -2.10993500 -5.64033400
	F -3.87649400 -2.35788300 -4.41137500
	C 0.93122200 2.65909700 -2.86291100
	C 1.49692700 3.53420200 -3.78118400
	C -0.68508600 4.04825100 -4.63033700
	C -1.24813200 3.17871800 -3.71573900
	C -0.44229500 2.47880000 -2.82594900
	O -1.05530800 1.63015000 -1.95050800
	H 1.57201000 2.11566200 -2.17481000
	H 2.57448500 3.66776300 -3.80040900
	H -1.30659100 4.59671400 -5.32775300
	H -2.32072300 3.03059900 -3.68144100
	H -0.39322700 1.22210600 -1.38643100
	C 0.69246600 4.23250800 -4.66926500
	O 1.18731300 5.10227200 -5.59150400
	H 2.14431400 5.13591100 -5.51606400
	Cl -2.41393700 -0.37039200 -3.81324600
Br…O	C -3.25637000 -1.68854200 -4.99066900
	Br -2.45760600 -0.39006900 -3.80964900
	F -4.20221400 -1.12449800 -5.72534700
	F -2.34302600 -2.19951000 -5.80162400
	F -3.79831700 -2.67588600 -4.29420900
	C 0.96115800 2.71306100 -2.86186300
	C 1.55543200 3.61478500 -3.73512300
	C -0.59846000 4.15250200 -4.64051700
	C -1.19007600 3.25654200 -3.77057400
	C -0.41232500 2.53215800 -2.87594400
	O -1.05297300 1.65766200 -2.04475500
	H 1.57953000 2.14948200 -2.16953900
	H 2.63283000 3.74918700 -3.71517900
	H -1.19735800 4.72059900 -5.34193300
	H -2.26306500 3.10706500 -3.77594000
	H -0.40950600 1.23255800 -1.47159500
	C 0.77942300 4.33834900 -4.62845100
	O 1.30286700 5.23463700 -5.50797200
	H 2.25695500 5.26631200 -5.40106100

I…O	C	-3.46083600	-1.98266300	-4.87713600
	I	-2.43696700	-0.47377000	-3.74157100
	F	-3.86511200	-1.49867500	-6.04513300
	F	-2.66245600	-3.01731900	-5.11183500
	F	-4.52706800	-2.42397900	-4.22078500
	C	1.07151600	2.77937400	-2.97032700
	C	1.61143200	3.75068900	-3.80306700
	C	-0.58437400	4.27103200	-4.61484600
	C	-1.12222800	3.30649000	-3.78423400
	C	-0.29525500	2.55724000	-2.95790200
	O	-0.88360000	1.61211800	-2.16157200
	H	1.72687700	2.19478100	-2.33163700
	H	2.68422200	3.91853900	-3.80501600
	H	-1.22082800	4.85990100	-5.26400400
	H	-2.19005100	3.12391800	-3.76841400
	H	-0.21264200	1.18505400	-1.62194500
	C	0.78702400	4.50039600	-4.62932200
	O	1.25580800	5.46351100	-5.46671500
	H	2.21143400	5.51975900	-5.38648400
Cl…N	C	-2.76579800	-1.53229500	-4.14257600
	Cl	-1.89172700	-0.01526400	-4.09772400
	F	-3.91788500	-1.38733900	-4.78185700
	F	-2.05570900	-2.46070800	-4.76773100
	F	-3.02148100	-1.95969800	-2.91429700
	C	0.94374500	2.65115500	-4.01084800
	C	1.63551100	3.85487200	-3.97738200
	C	-0.31667700	4.97863100	-3.96932600
	C	-1.01034700	3.77602200	-4.00279200
	H	1.47200300	1.70237400	-4.02767900
	H	2.72139000	3.87225200	-3.96730700
	H	-0.84692000	5.92631200	-3.95258400
	H	-2.09618000	3.75637700	-4.01297400
	N	-0.38215200	2.60769300	-4.02366700
	N	1.00936300	5.02456700	-3.95644100
Br…N	C	-2.80770400	-1.60497300	-4.14459300
	Br	-1.84414400	0.06782000	-4.09614200
	F	-3.96029900	-1.46064000	-4.78355100
	F	-2.09783600	-2.53385800	-4.76976400
	F	-3.06308400	-2.03237700	-2.91599900
	C	0.95698100	2.67152000	-4.01042400
	C	1.64791600	3.87556200	-3.97659900
	C	-0.30500700	4.99970100	-3.96858500
	C	-0.99933400	3.79762200	-4.00240400
	H	1.48356000	1.72199200	-4.02739600
	H	2.73370600	3.89341100	-3.96635300
	H	-0.83483500	5.94752000	-3.95171100
	H	-2.08497100	3.77611600	-4.01277500
	N	-0.36878500	2.63081300	-4.02337100
	N	1.02097000	5.04472400	-3.95551800
I…N	C	-2.86928800	-1.71292900	-4.14833800
	I	-1.79354800	0.15434500	-4.09216800
	F	-4.02757800	-1.57633000	-4.78817000
	F	-2.16393900	-2.65040000	-4.77549700
	F	-3.12995300	-2.15112600	-2.91978800
	C	0.98044500	2.70808300	-4.00750400
	C	1.66969700	3.91284700	-3.97523400
	C	-0.28465600	5.03729700	-3.96868500
	C	-0.97994000	3.83600800	-4.00094300
	H	1.50418600	1.75727900	-4.02333600
	H	2.75529300	3.93132600	-3.96504500
	H	-0.81422600	5.98507000	-3.95307800
	H	-2.06522300	3.81097500	-4.01139400
	N	-0.34540100	2.67138000	-4.02029500
	N	1.04126400	5.08112600	-3.95571100
Bifurcated				
Cl…O/O	C	-0.47938400	12.22595700	4.15629800
	C	0.63524200	11.41641800	4.30165900
	C	1.73146300	11.59883100	3.45112300
	C	1.68245100	12.58533700	2.48015400
	H	2.53739100	12.71864000	1.82312500
	C	0.56188500	13.39432500	2.34057800
	H	0.54393000	14.15974100	1.57442900
	C	-0.52189500	13.21395200	3.18080100
	H	-1.40352000	13.83571500	3.08410400
	O	0.73136400	10.43442100	5.23172300

	H -0.09521500 10.38293300 5.71915400<br>O 2.79547900 10.77619700 3.62926100<br>Cl 1.14573300 8.09399000 2.91373600<br>H 3.46126600 10.97798900 2.96624600<br>H -1.32680400 12.07688500 4.81963300<br>C 0.43746200 6.87806800 1.87545600<br>F 0.87319800 5.67138000 2.20632900<br>F 0.75631600 7.10446700 0.60923900<br>F -0.88346200 6.88856500 1.98144000
Br…O/O	C -0.46833700 12.39880600 4.20267900<br>C 0.58219500 11.49655500 4.20119000<br>C 1.66455000 11.70168800 3.33875800<br>C 1.66640200 12.80443800 2.50065900<br>H 2.51009300 12.95607000 1.83327000<br>C 0.60982200 13.70621700 2.50706400<br>H 0.63042100 14.56208000 1.84355200<br>C -0.45998500 13.50313700 3.36000800<br>H -1.29141500 14.19710900 3.37584400<br>O 0.62827500 10.39818500 4.99718500<br>H -0.18718800 10.34116600 5.50261300<br>O 2.66367800 10.78540500 3.37568700<br>Br 0.96689600 7.92839100 2.93880200<br>H 3.31610200 11.00324700 2.70456800<br>H -1.30529800 12.23050800 4.87454900<br>C 0.49895500 6.38451100 1.88753800<br>F 0.84736400 5.27146900 2.51536300<br>F 1.11770900 6.41503400 0.71629600<br>F -0.80733700 6.34979600 1.66886400
I…O/O	C -0.46838800 12.57786600 4.23534200<br>C 0.48879100 11.59039300 4.07712100<br>C 1.57252100 11.81025000 3.22125900<br>C 1.67108000 13.01439000 2.54432400<br>H 2.51532100 13.17769800 1.88057400<br>C 0.70816800 14.00223600 2.70707400<br>H 0.80257700 14.93739700 2.16886400<br>C -0.36349200 13.78394900 3.55409200<br>H -1.12193300 14.54506000 3.69005900<br>O 0.44127800 10.39006900 4.71184000<br>H -0.37018300 10.33270000 5.22388000<br>O 2.47537400 10.80481500 3.10446000<br>I 0.88995800 7.77214900 2.86683800<br>H 3.13399000 11.04279500 2.44628400<br>H -1.30736200 12.39698800 4.90125800<br>C 0.58006400 5.87694200 1.91102100<br>F 0.99185400 4.88223500 2.68859800<br>F 1.25069300 5.80962400 0.76627500<br>F -0.70741000 5.68625800 1.64532400
Cl…N/N	C -8.19918200 -3.16773500 0.05826900<br>C -6.83218400 -3.26130600 0.04420900<br>C -6.06041800 -2.08893300 0.02019300<br>C -6.74703900 -0.85325600 0.01168400<br>C -8.77776900 -1.89062000 0.04791200<br>C -4.63403400 -2.11927300 0.00458900<br>C -5.97858900 0.37467900 -0.01288100<br>C -4.56715500 0.29656300 -0.02771400<br>C -3.91674000 -0.97331800 -0.01847200<br>C -3.84985200 1.50303900 -0.05142800<br>H -2.76496300 1.47818600 -0.06330800<br>C -4.53161100 2.69161500 -0.05913200<br>C -5.93327600 2.65394900 -0.04286200<br>H -4.13662200 -3.08351700 0.01161900<br>H -8.82680500 -4.05044500 0.07690200<br>H -6.33570700 -4.22629200 0.05131300<br>H -9.86006300 -1.78366000 0.05856300<br>H -2.83210600 -0.99953300 -0.03032200<br>H -4.01198400 3.64195600 -0.07726600<br>H -6.50199200 3.58096700 -0.04837900<br>N -8.08628200 -0.77562400 0.02555000<br>N -6.63434000 1.54492900 -0.02065600<br>C -11.32150100 2.77867600 -0.00141200<br>F -12.12261900 2.43115500 0.99970500<br>F -11.03533300 4.06941800 0.13139300<br>F -11.99223100 2.62201000 -1.13720400<br>Cl -9.86864700 1.81621200 0.00115600
Br…N/N	C -8.21474200 -3.11287800 -0.00510400

	C	-6.84955800	-3.23246200	-0.00378300
	C	-6.05441700	-2.07549700	-0.00293200
	C	-6.71600500	-0.82658700	-0.00345100
	C	-8.76876400	-1.82533700	-0.00548200
	C	-4.62874100	-2.13279600	-0.00164900
	C	-5.92503200	0.38642000	-0.00276300
	C	-4.51551500	0.28179200	-0.00164000
	C	-3.88967400	-1.00042400	-0.00104100
	C	-3.77634700	1.47528900	-0.00122200
	H	-2.69203900	1.43109600	-0.00035400
	C	-4.43661700	2.67600900	-0.00197600
	C	-5.83893500	2.66434800	-0.00305000
	H	-4.14981000	-3.10631400	-0.00121500
	H	-8.85934400	-3.98334700	-0.00585200
	H	-6.37219400	-4.20703000	-0.00339900
	H	-9.84845100	-1.69394400	-0.00652300
	H	-2.80570100	-1.04740100	-0.00014600
	H	-3.89959900	3.61678700	-0.00173000
	H	-6.39082300	3.60134700	-0.00369500
	N	-8.05355700	-0.72526600	-0.00467200
	N	-6.55972000	1.56788700	-0.00341900
	C	-11.50542800	2.75189800	0.00361000
	F	-11.94892000	2.94056200	1.24136900
	F	-11.26924200	3.94364300	-0.53392500
	F	-12.47488700	2.17163200	-0.69564200
	Br	-9.91498400	1.67041800	0.00170900
I…N/N	C	-8.18558700	-3.13511800	-0.00628300
	C	-6.82020400	-3.25394000	-0.00620900
	C	-6.02516800	-2.09693900	-0.00451300
	C	-6.68680700	-0.84865700	-0.00289200
	C	-8.74122200	-1.84888000	-0.00456800
	C	-4.59928300	-2.15196400	-0.00443100
	C	-5.89765200	0.36406500	-0.00127100
	C	-4.48854700	0.26285700	-0.00136900
	C	-3.86135100	-1.01868100	-0.00291500
	C	-3.75365000	1.45911700	0.00002500
	H	-2.66924800	1.41886100	0.00000000
	C	-4.41779100	2.65793500	0.00129300
	C	-5.82001000	2.64317000	0.00120000
	H	-4.11911800	-3.12478200	-0.00567100
	H	-8.82981000	-4.00571600	-0.00759100
	H	-6.34278600	-4.22840600	-0.00744700
	H	-9.82060400	-1.71552000	-0.00450700
	H	-2.77740300	-1.06479200	-0.00292500
	H	-3.88344500	3.60015400	0.00230600
	H	-6.37646300	3.57737500	0.00211800
	N	-8.02472800	-0.74927500	-0.00292200
	N	-6.53510000	1.54321900	0.00002900
	C	-11.65198400	2.86278200	0.00207800
	F	-12.08637900	3.08412500	1.24227800
	F	-11.43683300	4.04883600	-0.56685600
	F	-12.63748800	2.26775400	-0.66955500
	I	-9.87038600	1.66226300	0.00262400
